# Estimating Global Prevalence of Metabolic Dysfunction-Associated Fatty Liver Disease in Overweight or Obese Children and Adolescents: Systematic Review and Meta-Analysis

**DOI:** 10.3389/ijph.2021.1604371

**Published:** 2021-10-06

**Authors:** Jiaye Liu, Chunyang Mu, Kewei Li, Han Luo, Yong Liu, Zhihui Li

**Affiliations:** ^1^ Department of Thyroid and Parathyroid Surgery, West China Hospital, Sichuan University, Chengdu, China; ^2^ Laboratory of Thyroid and Parathyroid Diseases, Frontiers Science Center for Disease-Related Molecular Network, West China Hospital, Sichuan University, Chengdu, China; ^3^ Department of Liver Surgery and Liver Transplantation Center, West China Hospital, Sichuan University, Chengdu, China; ^4^ Department of Pediatrics, West China Hospital, Sichuan University, Chengdu, China; ^5^ Department of Gastroenterological Surgery, West China Hospital, Sichuan University, Chengdu, China

**Keywords:** children, prevalence, adolescent, metabolic dysfunction-associated fatty liver disease, non-alcoholic fatty liver disease

## Abstract

**Objectives:** Metabolic dysfunction-associated fatty liver disease (MAFLD) is a new terminology updated from non-alcoholic fatty liver disease (NAFLD). We aim to estimate the global prevalence of MAFLD in overweight or obese children and adolescents, by repurposing existing data on fatty liver disease.

**Methods:** We screened relevant articles published up to December 2020. Pooled prevalence was calculated using Logit transformations.

**Results:** Our search returned 35,441 records, of which 156 studies fulfilled the inclusion criteria. The overall prevalence of MAFLD was 33.78% in the general population and 44.94% in a special population based on child obesity clinics, regardless of the diagnostic techniques. For subgroup analysis, MAFLD prevalence was significantly higher in boys compared to girls (36.05 vs. 26.84% in the general population; 50.20 vs. 35.34% in the child obesity clinics-based population). Interestingly, based on study source, the pooled prevalence of MAFLD was 1.5-fold higher in other “fatty liver disease” studies compared to the classical “NAFLD” studies in the general population.

**Conclusion:** MAFLD is highly prevalent in overweight or obese children and adolescents. Raising awareness and urgent actions are warranted to control the MAFLD pandemic across the globe.

## Introduction

Obesity has become a global pandemic, but the growing burden in the pediatric population is even more worrisome [1]. Approximately 400 million children and adolescents were estimated to be overweight or obese in 2016 [2], which has vital short- and long-term health consequences. These children are more likely to suffer from psychological comorbidities, cardiovascular disease, diabetes, cancers, musculoskeletal problems, and liver complications [3–5].

Fatty liver disease has emerged as one of the most common comorbidities in the pediatric obese population [6]. Previous studies have documented that the prevalence of non-alcoholic fatty liver disease (NAFLD) in the pediatric population ranges from 3 to 12%, but this rate reaches as high as 70–80% in obese children [7, 8]. However, the classical terminology of NAFLD has recently been redefined and is now referred to as metabolic dysfunction-associated fatty liver disease (MAFLD) [9, 10]. This revised nomenclature is expected to have an impact on advancing disease diagnosis, patient management, therapeutic development, and public health in combating fatty liver disease. A key advance fostered by the concept of MAFLD is that the disease is defined using a set of positive criteria and, therefore, this is no more “exclusion” diagnoses [11, 12]. The diagnosis of MAFLD is based on the detection of hepatic steatosis by histology, imaging, or blood biomarkers in addition to one of the following three conditions: excess adiposity, presence of prediabetes or type 2 diabetes, or evidence of metabolic dysregulation [13].

This paradigm shift in disease definition prompts calls for a re-assessment of the burden of fatty liver disease. We hypothesized that a substantial proportion of existing data on fatty liver disease in overweight or obese populations can be repurposed to estimate MAFLD prevalence by applying the newly defined criteria. In this study, we performed a systematic review and meta-analysis to estimate the global prevalence of MAFLD in overweight and obese children and adolescents by exploring and repurposing available literature data of fatty liver disease.

## Methods

### Literature Search

A systematic search was conducted in Medline, Embase, Web of Science, Cochrane CENTRAL Databases, and Google scholar for articles in the English language up until December 2020. All searches were performed by a biomedical information specialist of the medical library, with an exhaustive set of search terms related to “non-alcoholic fatty liver disease”, “fatty liver”, “hepatic steatosis”, “prevalence”, and “epidemiology” (the full search strategies are provided in Supplementary Methods 1). We only included studies of which the source data presented in the article could be repurposed to calculate the MAFLD prevalence in overweight or obese children or adolescents.

### Inclusion and Exclusion Criteria

The diagnosis of MAFLD was in accordance with the recent consensus on the criteria of diagnosing MAFLD [9, 10, 13]. As proposed, MAFLD should be based on detection of hepatic steatosis in addition to one of the following three conditions, namely excess adiposity, presence of prediabetes or type 2 diabetes, or evidence of metabolic dysregulation. Because of the change in disease definition, to our knowledge, there is currently one published study on the global epidemiology of MAFLD by our group [14]. However, the MAFLD prevalence in children and adolescents is largely unknown. Studies were included if they fulfilled the following criteria: 1) children or adolescents aged from 1 to 19 years old; 2) the study provided adequate information on the prevalence of fatty liver disease; 3) the diagnostic technique can be transformed to the new criteria for diagnosing MAFLD (histological, imaging, or blood biomarker evidence of fat accumulation in the liver); 4) participants were either overweight or obese; and 5) study period from Jan, 1980 to Dec, 2020.

Studies were excluded if any of these criteria were unmet and/or: 1) studies were reviews, meta-analyses, abstracts, and letters or correspondence; 2) studies with incomplete data; 3) participants were not overweight or obese individuals; 4) studies were not written in English; and 5) fatty liver was diagnosed by elevated ALT or AST. Our analysis in this review is in accordance with PRISMA guidelines [15].

### Screening and Data Extraction

Studies were screened based on pre-specified decision rules. Initial title and abstract screening was done independently by two reviewers (JL, CM), with a random 10% of studies checked by two additional investigators (HL, YL). Full-text review was done independently by two authors (any two of JL, CM, HL, and YL), with discrepancies resolved by consensus or by a fifth reviewer (ZL); consensus was reached in all instances. We extracted data at all levels reported in the studies, including time of publication, study period, country or region, country or region income based on World Bank evaluation, the level of country development, study categories, gender, age, diagnostic techniques, body mass index (BMI), source of the studies, and prevalence of disease. Here source of studies was divided into two categories–“classical NAFLD” and “other fatty liver disease”. “Classical NAFLD” was in accordance with the diagnostic criteria of NAFLD. “Other fatty liver disease” in the current study mainly include fatty liver disease coexisting with hepatitis virus infection or unspecified fatty liver disease. Data were cross checked for accuracy against the original source by one of four authors (JL, CM, HL, or YL).

### Quality Assessment

Two authors (any two of JL, CM, HL, and YL) independently reviewed and extracted data from the included studies by using a data extraction form specifically designed for this study. When duplicate data were identified, the duplicate with the smallest sample size or shortest duration of follow-up was excluded. We assessed the quality of included studies using the Newcastle-Ottawa Scale, which is comprised of three domains; selection, comparability, and outcome. The Newcastle-Ottawa Scale assigns a maximum score of five for selection, two for comparability, and two for outcome [16]. Studies scoring 1–3 were defined as low, 4–6 as average, and 7–9 as high quality (Supplementary Table S1). Studies were not excluded on the basis of their quality score in order to increase transparency and to ensure that all available evidence of this topic was reported.

### Statistical Analysis

The “Meta”, ”Metafor”, and “Dmetar” modules in the R‐4.0.2 statistical software package were used for meta‐analysis. The main outcome for this study was the global MAFLD prevalence for overweight and obese children and adolescents. The diagnostic criteria of overweight and obese were defined by each original study. To calculate MAFLD prevalence for each country and region, we estimated the pooled rate by using the DerSimonian-Laird random-effects model with Logit transformations. Heterogeneity across the included studies was assessed using the Cochran Q statistics and I^2^ statistics. A random-effect model was used as high heterogeneity was frequently observed in the pooling prevalence meta-analysis. Sensitivity analysis was performed by using “Leave-one-out” analysis with a build-in function. After outliers were identified, we re-estimated the pooling prevalence by removing the outlying studies. Univariate meta-regression and multi-variable meta-regression were performed by using the “dmetar” package in R. P-value was used to compare the difference between subgroup analysis. Subgroup analysis was done to further explore the source of heterogeneity which estimated the pooled rate by dividing individuals into covariates. Egger’s test was used to assess publication bias.

## Results

### Study Characteristics

**FIGURE 1:**
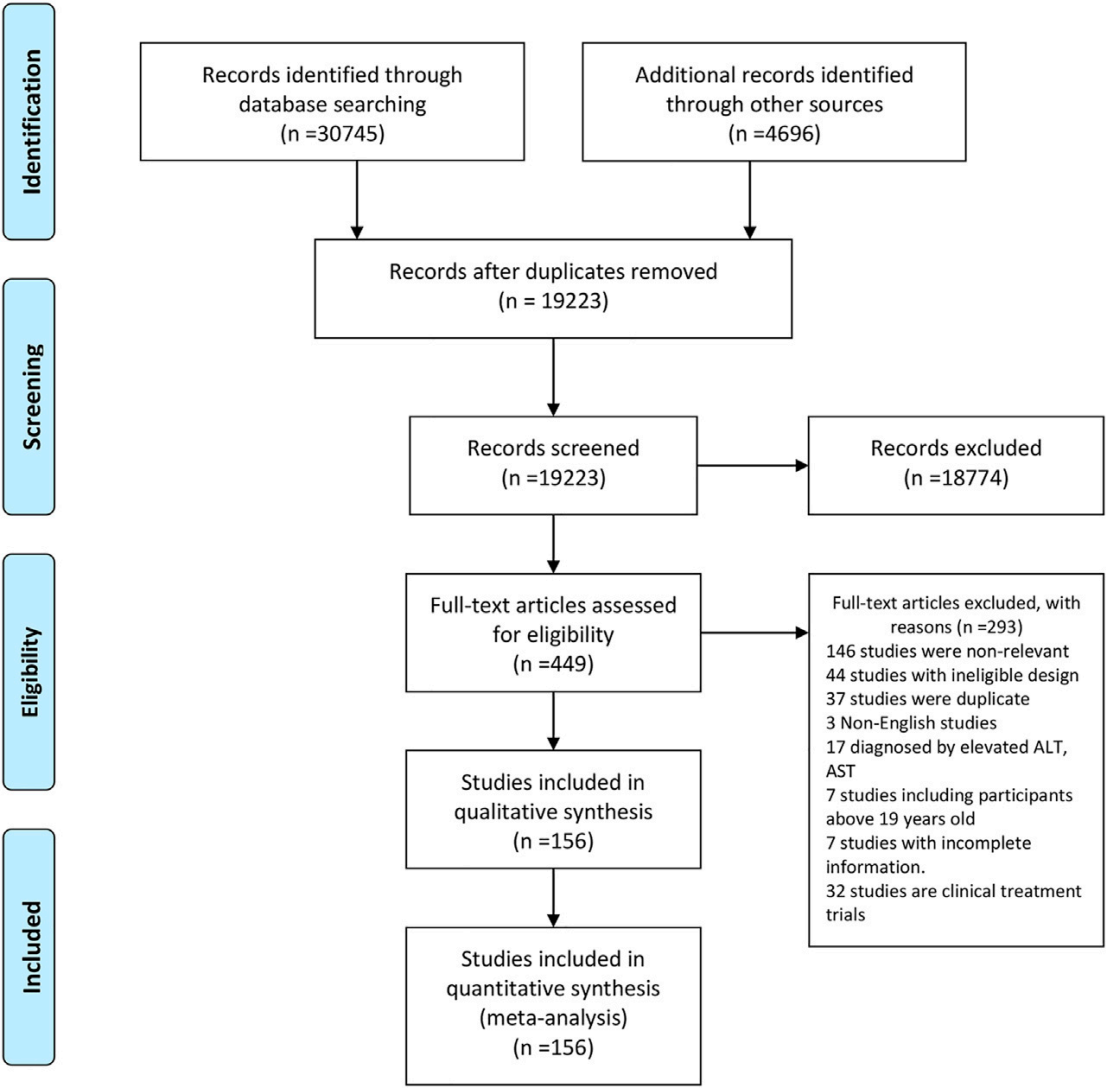
Study selection. West China Hospital Metabolic Dysfunction-Associated Fatty Liver Disease Project, China, 2020–2021.

Our search returned 35,441 records. After removing duplicates, 19,223 studies were retained. By screening titles and abstracts, 18,774 records were further excluded. Full text of the remaining 449 studies were assessed for eligibility of which 293 were excluded. Finally, 156 studies from 33 countries and regions [Albania (*n* = 1), Australia (*n* = 2), Brazil (*n* = 12), Mainland China (*n* = 12), Canada (*n* = 2), Chile (*n* = 1), Colombia (*n* = 2), Denmark (*n* = 3), Egypt (*n* = 4), Germany (*n* = 5), Germany, Austria, and Switzerland (*n* = 1), Greece (*n* = 2), Hong Kong (*n* = 1), India (*n* = 5), Iran (*n* = 8), Israel (*n* = 2), Italy (*n* = 15), Japan (*n* = 3), Malaysia (*n* = 1), Mexico (*n* = 2), Netherlands (*n* = 4), Pakistan (*n* = 1), Poland (*n* = 5), Romania (*n* = 2), Saudi Arabia (*n* = 1), South Korea (*n* = 7), Spain (*n* = 6), Taiwan (*n* = 8), Sri Lanka (*n* = 1), Turkey (*n* = 22), United Arab Emirates (*n* = 1), and United States (*n* = 14)] fulfilled our inclusion criteria (Figure 1). The quality assessment score for included studies ranged from 6 to 9, with a mean quality score of 7.44. A total of 147 high-quality and 9 fair-quality studies were included in the meta-analysis (Supplementary Table S1). Characteristics of all included studies are listed in Supplementary Table S1. The majority of studies had a cross-sectional design and most of them reported data from hospital or outpatient clinic settings. The mean or median age of participants across different studies ranged from 7.00 to 17.01 years, and the percentages of boys ranged from 19.60 to 100%.

### MAFLD Prevalence in Overweight or Obese Children and Adolescents from the General Population

**FIGURE 2:**
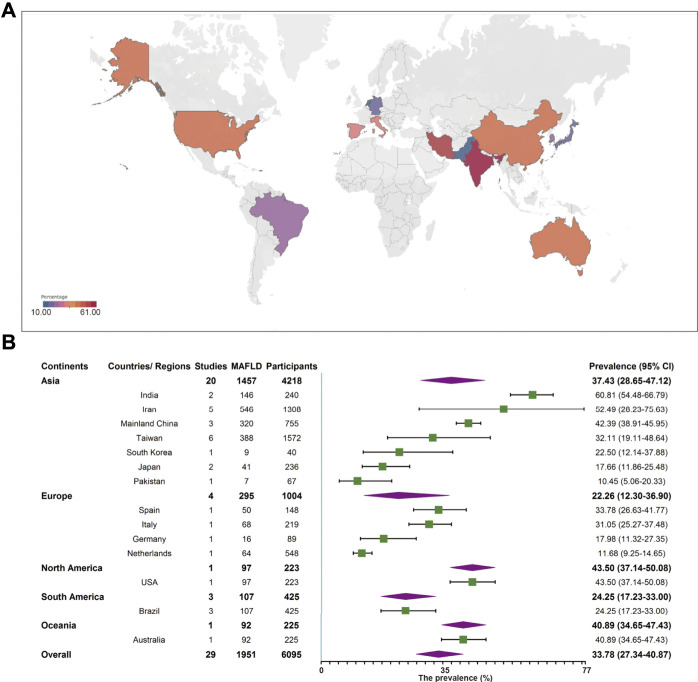
Metabolic dysfunction-associated fatty liver disease prevalence in the general population regardless of the diagnostic techniques. **(A)** Forest plot of metabolic dysfunction-associated fatty liver disease prevalence. **(B)** Metabolic dysfunction-associated fatty liver disease prevalence in 14 countries and regions. West China Hospital Metabolic Dysfunction-Associated Fatty Liver Disease Project, China, 2020–2021.

Among the included studies, 29 studies comprising 6,095 individuals reported MAFLD prevalence in overweight or obese children and adolescents from the general population. The overall prevalence in this population was 33.78% (95% CI 27.34–40.87, Figure 2) regardless of diagnostic techniques. By performing sensitivity analysis, one outlier was identified (Supplementary Tables S2, S3, Supplementary Figures S1, S2). After removing it, the prevalence slightly decreased to 31.71% (95% CI 25.86–38.20). Univariate meta-regression indicated that continents (R^2^ = 0, *p* = 0.61), country development (R^2^ = 0.01, *p* = 0.12), country or regional income (R^2^ = 0.17, *p* = 0.34), publication time (R^2^ = 0, *p* = 0.19), and quality score (R^2^ = 0.17, *p* = 0.34, Supplementary Table S4) were not significantly correlated with the high heterogeneity. Multi-variable meta-regression revealed that country development had the highest predictor importance (32.54%, Supplementary Figure S5 and Supplementary Table S5). By stratifying data according to continents, the prevalence of MAFLD was 43.50% (95% CI 37.14–50.08), 40.89% (95% CI 34.65–47.43), 37.43% (95% CI 28.65–47.12), 24.25% (95% CI 17.23–33.00), and 22.26% (95% CI 12.30–36.90) in North America, Oceania, Asia, Europe, and South America, respectively. The highest rate was observed in India (60.81%, 95% CI 54.48–66.79) and the lowest in Pakistan (10.45%, 95% 5.06–20.33, Figure 2). The majority of studies (89.66%) used ultrasound to diagnose MAFLD with a pooled prevalence rate of 34.16% (95% CI 27.46–41.55, Table 1). Interestingly, our results suggest that MAFLD was more prevalent in developing countries (34.16%, 95% CI 27.46–41.55) than developed countries (30.72%, 95% CI 11.00–61.39, *p* = 0.05, Table 1). With respect to the income of countries or regions, the pooled estimate prevalence of high, upper-middle, and lower-middle income countries or regions was 27.56% (95% 20.75–35.60), 41.13% (95% CI 30.12–53.11), and 39.00% (95% CI 13.98–71.55, Table 1). Moreover, MAFLD prevalence was 27.32% (95% CI 20.88–34.87) in participants below 10 years old and 42.59% (95% CI 27.41–59.31, Table 1) in those above 10 years old. The pooled prevalence was 36.05% (95% CI 24.68–49.23) and 26.84% (95% CI 19.46–35.79, Table 1) in boys and girls, respectively. When stratifying participants by BMI, MAFLD prevalence was 20.23% (95% CI 12.87–30.33) in overweight participants and 38.47% (95% CI 29.75–48.00, *p* = 0.01, Table 1) in obese participants. Eighteen classical “NAFLD” studies and eight other “fatty liver disease” studies generated a pooled MAFLD prevalence of 30.80% (95% CI 24.25–38.24) and 43.13% (95% CI 24.39–64.07, *p* = 0.25, Table 1), respectively, in the general population.

**TABLE 1:** Subgroup analysis of metabolic dysfunction-associated fatty liver disease prevalence in the general population and in the special population based on child obesity clinics. West China Hospital Metabolic Dysfunction-Associated Fatty Liver Disease Project, China, 2020–2021.

	General population				Special population			
	Studies	Prevalence (95% CI)	I^2^	P	Studies	Prevalence (95% CI)	I^2^	P
Continents			96%	<0.01			98%	<0.01
Asia	20	37.43% (28.65–47.12)	97%		53	49.66% (43.84–55.49)	97%	
Europe	4	22.26% (12.30–36.90)	95%		40	38.27% (30.86–46.26)	99%	
Oceania	1	40.89% (34.65–47.43)	-		1	67.44% (58.90–74.96)	-	
Africa	-	-	-		4	52.21% (34.61–69.29)	86%	
North America	1	43.50% (37.14–50.08)	-		17	46.22% (32.78–60.22)	98%	
South America	3	24.25% (17.23–33.00)	71%		12	39.28% (31.00–48.24)	87%	
Development			96%	0.05			98%	0.10
Developed	9	25.13% (17.25–35.07)	94%		62	41.23% (34.95–47.81)	98%	
Developing	20	38.19% (29.62–47.55)	97%		65	48.24% (43.15–53.36)	96%	
Income			91%	0.14			98%	0.54
High	15	27.56% (20.75–35.60)	95%		67	42.59% (36.39–49.03)	98%	
Upper-middle	11	41.13% (30.12–53.11)	95%		54	47.23% (41.89–52.64)	96%	
Lower-middle	3	39.00% (13.98–71.55)	95%		6	47.89% (29.22–67.17)	97%	
Gender			89%	0.22			92%	<0.01
Boy	9	36.05% (24.68–49.23)	91%		30	50.20% (45.34–55.05)	89%	
Girl	9	26.84% (19.46–35.79)	83%		30	35.34% (30.46–40.55)	90%	
Age			97%	0.08			99%	0.42
1< and ≤ 10 years	4	27.32% (20.88–34.87)	62%		4	33.40% (16.86–55.36)	95%	
10 < and ≤ 19 years	10	42.59% (27.41–59.31)	97%		13	45.43% (26.45–65.83)	99%	
Publication time			96%	0.06			98%	0.99
Before 2010	10	25.18% (16.38–36.63)	94%		28	44.77% (32.60–57.61)	99%	
After 2010	19	38.52% (30.26–47.50)	97%		99	44.72% (41.01–48.49)	96%	
Study period			96%	0.11			98%	0.64
Before 2010	17	25.77% (19.44–33.31)	94%		42	44.18% (34.64–54.18)	99%	
After 2010	10	49.06% (36.43–61.80)	96%		76	44.72% (39.94–49.60)	96%	
Sample size			96%	0.71			98%	<0.01
<100	8	31.38% (18.24–48.39)	91%		48	52.78% (47.14–58.35)	87%	
≥100	21	34.68% (27.26–42.94)	97%		79	40.30% (35.06–45.77)	99%	
Quality			96%	0.56			98%	0.45
<8	12	36.08 (24.31–49.79)	94%		70	46.25% (41.58–50.99)	95%	
≥8	17	31.78% (25.23–39.13)	97%		57	42.95% (35.95–50.25)	99%	
Diagnostic method			96%	0.81			98%	<0.01
Ultrasound	26	34.16% (27.46–41.55)	96%		96	46.44% (41.33–51.63)	98%	
MRI	3	30.72% (11.00–61.39)	98%		15	34.33% (29.62–39.36)	84%	
Biopsy					9	44.81% (18.76–74.07)	99%	
Fatty liver score					1	22.11% (16.88–28.41)	-	
H-MRS					5	50.13% (30.82–69.40)	91%	
CAP					1	53.17% (44.45–61.71)	-	
BMI			95%	<0.01			98%	<0.01
Overweight	11	20.23% (12.87–30.33)	93%		11	25.13% (14.75–39.43)	97%	
Obese	20	38.47% (29.75–48.00)	95%		95	46.01% (40.50–51.62)	98%	
Study source			96%	0.09			98%	0.97
Classical “NAFLD” studies	18	29.16% (22.24–37.19)	96%		110	44.83% (40.11–49.65)	98%	
Other “fatty liver disease” studies	11	42.48% (29.45–56.65)	96%		17	44.61% (34.55–55.13)	95%	

BMI, body mass index.

### MAFLD Prevalence in Overweight or Obese Children and Adolescents From Child Obesity Clinics

**FIGURE 3:**
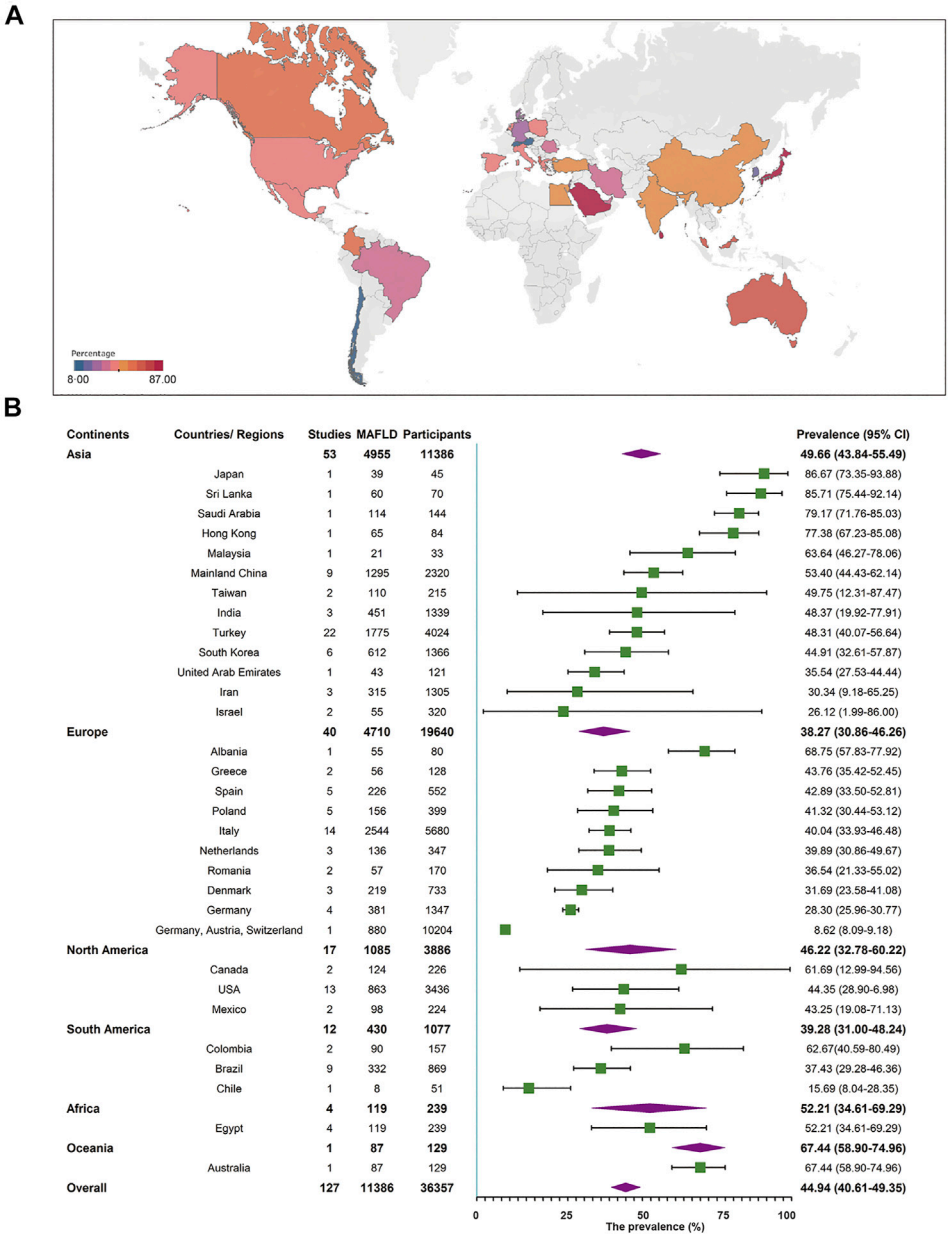
Metabolic dysfunction-associated fatty liver disease prevalence in the special population based on child obesity clinics regardless of the diagnostic techniques. **(A)** Forest plot of metabolic dysfunction-associated fatty liver disease prevalence. **(B)** Metabolic dysfunction-associated fatty liver disease prevalence in 32 countries and regions. West China Hospital Metabolic Dysfunction-Associated Fatty Liver Disease Project, China, 2020–2021.

A total of 127 studies comprising 36,357 individuals were included for estimating MAFLD prevalence in overweight and obese patients based on studies from child obesity clinics. The overall prevalence rate, regardless of the diagnostic technique used, was 44.81% (95% CI 40.46–49.23, Figure 3), with high heterogeneity observed. After removing the outlier, the prevalence increased to 45.40% (95% CI 41.15–49.73, Supplementary Figures S3, S4, Supplementary Tables S6, S7). Both univariate and multi-variable meta-regression suggest that study size has a high predictor importance accounting for the high heterogeneity (Supplementary Figure S6 and Supplementary Tables S8, S9). For subgroup analysis, the pooled regional prevalence estimates in this population were 49.66% (95% CI 43.84–55.49) for Asia, 38.27% (95% CI 30.86–46.26) for Europe, 52.21% (95% CI 34.61–69.29) for Africa, 46.22% (95% CI 32.78–60.22) for North America, 39.28% (95% CI 31.00–48.24) for South America, and 67.44% (95% CI 58.90–74.94, Figure 3) for Oceania, though the number of studies for Oceania was limited. The prevalence varied substantially among different countries and regions, from 8.62% (Germany, Austria, and Switzerland, 95% CI 8.09–9.18) to 86.67% (Japan, 95% CI 73.35–93.88). For countries or regions with more than three studies included, MAFLD was most prevalent in Mainland China (53.40%, 95% CI 44.43–62.14) and the least in Germany (28.30%, 95% CI 25.96–30.77, Figure 3). Considering diagnostic techniques, more than three quarters of studies used ultrasound with a prevalence of 46.44% (95% CI 41.33–51.63, Table 1). There was a comparable prevalence of MAFLD between developing and developed countries (48.24% vs. 41.23, *p* = 0.10, Table 1). The pooled estimates among high, upper-middle, and lower-middle income countries or regions were 42.59% (95% CI 36.39–49.03), 47.23% (95% CI 41.89–52.64), and 47.89% (95% CI 29.22–67.17, Table 1), respectively. Patients aged above 10 years old had a slightly higher prevalence rate (45.43%) compared to those below 10 years old (33.40%), although without a statistical significant difference (*p* = 0.42, Table 1). The prevalence was significantly higher in boys (50.20%, 95% CI 45.34–55.05) than in girls (35.34%, 95% CI 30.46–40.55, Table 1). As expected, the pooled estimates revealed a significantly lower prevalence of 25.13% (95% CI 14.75–39.43) in overweight patients than obese ones (46.01%, 95% CI 40.50–51.62, *p* = 0.01, Table 1). There was little difference of MAFLD prevalence when comparing study period before and after the year 2010 (44.18 vs. 44.72%, *p* = 0.64, Table 1). MAFLD prevalence for studies with a quality assessment score above or below 8 points was 42.95% (95% CI 35.95–50.25) and 46.25% (95% CI 41.58–50.99, Table 1), respectively. Studies with a sample size less than 100 (52.78%, 95% 47.14–58.35) showed a higher prevalence rate than those with over 100 participants (40.30%, 95% CI 35.06–45.77, Table 1). Little difference was observed in MAFLD prevalence in this special population when comparing the classical “NAFLD” studies (44.83%, 95% CI 40.11–49.65) with other “fatty liver disease” studies (44.61%, 95% CI 34.55–55.13, Table 1).

The Egger’s plot for the prevalence data is depicted in Supplementary Table S10.

## Discussion

In this study, we performed a systematic review and meta-analysis based on existing epidemiology data on fatty liver disease in overweight or obese children and adolescents. These included the classical “NAFLD” and other “fatty liver disease” studies, and we transformed these existing literature data according to the new diagnosis criteria of MAFLD. We found a global MAFLD prevalence of 45% in settings based on child obesity clinics and 34% in the general population among overweight or obese children and adolescents aged between 1 and 19 years, regardless of the diagnostic technique used to establish the diagnosis. More importantly, we found an increased prevalence rate of 20.23% (95% CI 12.87–30.33) in overweight and 38.47% (95% CI 29.75–48.00) in obese children and adolescents within the general population. These rates were 25.13% (95% CI 14.75–39.43) and 46.01% (95% CI 40.50–51.62) in the clinical setting for overweight or obese children and adolescents, respectively.

The terminology NAFLD and non-alcoholic steatohepatitis (NASH) was described 40 years ago [17]. This definition requires the exclusion of other causes of liver diseases and of a daily consumption of a harmful amount of alcohol but the exact cut-off to define such an amount remains a debate. Because of this ambiguity, it was increasingly recognized that there was a need of redefining fatty liver disease [18]. MAFLD is a new terminology recently proposed by a panel of international experts to replace NAFLD [9, 10]. This updated nomenclature shifts attention towards an inclusionary diagnosis that does not require the exclusion of alcohol intake or other liver diseases. As proposed, MAFLD should be based on detection of liver steatosis with one of the following three conditions, including excess adiposity, presence of prediabetes or type 2 diabetes, or evidence of metabolic dysregulation [9, 10, 13]. A recent study pioneered to compare the differences between MAFLD and NAFLD criteria in real world settings using the large population-based National Health and Nutrition Examination Survey (NHANES) database. Although the diagnosis of MAFLD does not require the exclusion of alcohol consumption or other liver diseases, similar prevalence rates to NAFLD were detected. However, they found that the MAFLD criteria are more practical for identifying fatty liver disease patients with a high risk of disease progression [19]. Besides, another study also confirmed that the proposed criteria for diagnosis of MAFLD is well validated and easily applicable to the entire spectrum [20]. There are strong indications of global acceptance and endorsement for the term MAFLD [21–24], but there are also hot debates and skepticism. For example, there are valid arguments that currently there is no general consensus on the criteria to define ‘‘metabolic dysfunction”, and adding the new term “metabolic” does not fully solve the ambiguity regarding etiologies of the disease [25–27]. We noticed that MAFLD is relatively straightforward to define in the overweight or obese population. Globally, over 1.9 billion adults and about 400 million children and adolescents were overweight or obese in 2016 [2].

The prevalence of obesity has nearly tripled since 1975, and this parallels the growth of the fatty liver disease epidemic. Strong associations between obesity and prevalence of fatty liver disease have been well documented, including in children and adolescents [28]. A previous study attempted to depict the dynamic changes of NAFLD prevalence in adolescents aged 12–19 years using data from different periods of the NHANES database. Astonishingly, the prevalence of NAFLD has substantially risen from 3.9% in 1988–1994 to 10.7% in 2007–2010 [29]. A recent meta-analysis estimated that the NAFLD prevalence rate in overweight or obese children from the general population was 12.5 and 36%, respectively. NAFLD prevalence among children based on child obesity clinics has been reported to be 34% [8], but we observed a much higher (1.5 fold) rate of MAFLD in this population. This discrepancy may be attributed to differences in disease definition between NALFD and MAFLD, and/or population selection. Based on the source of included studies, we found that the pooled prevalence of MAFLD was 1.5-fold higher in other “fatty liver disease” studies compared to the classical “NAFLD” studies in the general population, but there was little difference in the special population from child obesity clinics. NAFLD could represent an umbrella term for multiple underlying sub-types which underestimates the prevalence of fatty liver disease [30, 31]. This is evidenced by the diagnostic criteria of NAFLD which excluded those with excessive alcohol intake as well as those with other causes of fatty diseases. The heterogeneity of the population with NAFLD, with respect to its primary driver and coexisting disease modifiers, impeded the progress of therapeutic development.

Extensive studies have highlighted the importance of techniques when diagnosing fatty liver disease. Fatty liver disease prevalence varies depending on the diagnostic techniques employed. If characterized by aspartate aminotransferase (AST) or alanine aminotransferase (ALT) values (>50 IU/L), estimates for children (aged 3–18 years) in Europe vary from 13 to 22.5% [32]. Fatty liver disease is predicted to affect 45% of obese teenagers in China [33]. Fatty liver disease, characterized as being overweight (BMI 95th percentile) with high ALT values, is predicted to affect 9.6% of all children aged 2–19 years in the United States, and 38.0% of children with obesity [34]. Ultrasound was the most commonly used diagnostic method in both clinical and general populations attributing to its satisfactory sensitivity and specificity [35, 36]. In the general population, we found no evidence of differences in MAFLD prevalence among different diagnostic techniques. In the clinical population, the prevalence of MAFLD diagnosed by H-MRS was higher than that of MRI or ultrasound. Interestingly, ultrasound and liver biopsy diagnoses yielded similar rates of MAFLD prevalence. Arguments have been raised against the application of liver enzymes for diagnosing fatty liver disease because normal levels of these enzymes have been widely observed in the entire spectrum of NAFLD [37, 38]. A recent study also documented that the accuracy of liver enzyme level for pediatric NAFLD diagnosis was 80% [39]. In line with this, the new diagnostic criteria of MAFLD no longer adopts elevated ALT and AST as markers for assessing fatty liver disease.

To our knowledge, this is a pioneering study to comprehensively estimate the global epidemiology of MAFLD. Nevertheless, some limitations of our study should be taken into consideration. Firstly, this study concerns a retrospective transformation of previous fatty liver disease data to MAFLD epidemiological estimation and by that we cannot exclude the possibility of inclusion bias. Secondly, limited data were available from Africa and Oceania that challenged the accuracy in estimating the prevalence rates for these continents. We noticed that MAFLD prevalence is extremely high in a special cohort in Africa. The situation may be even worse than our expectation, as viral hepatitis and HIV are widely spread in Africa which may augment MAFLD development. Thirdly, limited data regarding race, ethnicity, familial risk, epidemic factors, and genetic variation precluded us from performing further subgroup analysis. Fourthly, the high heterogeneity underlying some of the source data in the current study cannot be fully explained. Finally, there is no consistent standard for defining being overweight or obese across different countries and regions.

### Public Health Implications

This proof-of-concept study repurposed existing fatty liver disease data to estimate the prevalence and impact of MAFLD. It revealed a high prevalence rate of MAFLD in overweight or obese children and adolescents both in the general population and in the special population from child obesity clinics. These findings call for the awareness of the disease, attention of primary care physicians, specialists, and health policy makers to combat this disease. Future well-designed prospective studies are indicated to further define the global epidemiology and impact of MAFLD.
