# Supplementary material for: Estimating Global Prevalence of Metabolic Dysfunction-Associated Fatty Liver Disease in Overweight or Obese Children and Adolescents: Systematic Review and Meta-Analysis
Source: Int J Public Health. 2021 Oct 6;66:1604371. doi: 10.3389/ijph.2021.1604371 (PMC8527331; doi:10.3389/ijph.2021.1604371)
Supplement: Supplementary file 1 [file DataSheet1.DOCX]

**Supplementary file to**

**Estimating prevalence of metabolic dysfunction-associated fatty liver disease in overweight or obese children and adolescents**

**Supplementary methods 1. Searching strategy for metabolic dysfunction-associated fatty liver disease in overweight or obese children and adolescents.**

| **Database searched** | **Via** | **Records** | **Records after duplicates removed** |
| --- | --- | --- | --- |
| Embase | Embase.com | 11248 | 11075 |
| Medline ALL | Ovid | 10390 | 3157 |
| Web of Science Core Collection | Web of Knowledge | 8558 | 2260 |
| Cochrane Central Register of Controlled Trials | Wiley | 349 | 182 |
| Other sources: Google Scholar (200 top ranked) |  | 200 | 32 |
| Additional | By searching NAFLD | 4696 |  |
| Total |  | 35441 | 19223 |

**Embase**

('fatty liver'/exp OR 'metabolic liver disease'/de OR 'steatohepatitis'/de OR (hepatosteato* OR steatohepat* OR AFLD OR NAFLD OR FLD OR ((fatty OR steato*) NEAR/3 (liver OR hepat*)) OR ((metabol*) NEAR/3 (liver OR hepat*) NEAR/3 (diseas* OR syndrom*))):ab,ti,kw) AND ('epidemiological data'/de OR 'epidemiology'/de OR 'geographic distribution'/de OR 'patient volume'/de OR prevalence/exp OR geography/de OR 'geographic names'/exp OR 'cross-sectional study'/de OR (epidemiolog* OR ((geograph* OR global*) NEAR/3 (distribut*)) OR (patient* NEAR/3 volume*) OR prevalen* OR population-based* OR cross-sectional*):ab,ti,kw) NOT ([animals]/lim NOT [humans]/lim) NOT ('case report'/de OR 'case report*':ti) NOT ([Conference Abstract]/lim) AND [english]/lim

**Medline**

(exp Fatty Liver/ OR Liver Diseases/me OR (hepatosteato* OR steatohepat* OR AFLD OR NAFLD OR FLD OR ((fatty OR steato*) ADJ3 (liver OR hepat*)) OR ((metabol*) ADJ3 (liver OR hepat*) ADJ3 (diseas* OR syndrom*))).ab,ti,kw.) AND (Epidemiological Monitoring/ OR Epidemiology/ OR Epidemiology.fs. OR exp Incidence/ OR exp Prevalence/ OR Geography/ OR exp Geographic Locations/ OR Epidemiologic Studies OR Cross-Sectional Studies/ OR (epidemiolog* OR ((geograph* OR global*) ADJ3 (distribut*)) OR (patient* ADJ3 volume*) OR prevalen* OR population-based* OR cross-sectional*).ab,ti,kw.) NOT (exp Animals/ NOT Humans/) NOT (Case Reports/ OR case report*.ti.) NOT (news OR congres* OR abstract* OR book* OR chapter* OR dissertation abstract*).pt. AND english.la.

**Cochrane**

((hepatosteato* OR steatohepat* OR AFLD OR NAFLD OR FLD OR ((fatty OR steato*) NEAR/3 (liver OR hepat*)) OR ((metabol*) NEAR/3 (liver OR hepat*) NEAR/3 (diseas* OR syndrom*))):ab,ti) AND ((epidemiolog* OR ((geograph* OR global*) NEAR/3 (distribut*)) OR (patient* NEAR/3 volume*) OR prevalen* OR population-based* OR cross-sectional*):ab,ti)

**Web of Science**

TS=(((hepatosteato* OR steatohepat* OR AFLD OR NAFLD OR FLD OR ((fatty OR steato*) NEAR/2 (liver OR hepat*)) OR ((metabol*) NEAR/2 (liver OR hepat*) NEAR/2 (diseas* OR syndrom*)))) AND ((epidemiolog* OR ((geograph* OR global*) NEAR/2 (distribut*)) OR (patient* NEAR/2 volume*) OR prevalen* OR population-based* OR cross-sectional*)) NOT ((animal* OR rat OR rats OR mouse OR mice OR murine OR dog OR dogs OR canine OR cat OR cats OR feline OR rabbit OR cow OR cows OR bovine OR rodent* OR sheep OR ovine OR pig OR swine OR porcine OR veterinar* OR chick* OR zebrafish* OR baboon* OR nonhuman* OR primate* OR cattle* OR goose OR geese OR duck OR macaque* OR avian* OR bird* OR fish*) NOT (human* OR patient* OR women OR woman OR men OR man))) AND DT=(Article OR Review) AND LA=(English)

**Google Scholar**

hepatosteatosis|steatohepatitis|AFLD|NAFLD|FLD|"fatty|steatotic liver"|"liver|hepatic steatosis"|"metabolic liver disease" epidemiology|prevalence|"geographic|global distribution"|"patient volume"|"population based"|"cross sectional"

**Additional searching for Non-alcoholic fatty liver disease**

embase.com

('nonalcoholic fatty liver'/exp OR (((nonalcohol* OR non-alcohol*) NEAR/3 (fatty-liver* OR steatohepat* OR fld OR hepatosteato*)) OR ((nonalcohol* OR non-alcohol*) NEAR/3 steato* NEAR/3 (hepat* OR liver*)) OR nafld):ab,ti) AND ('epidemiological data'/de OR 'epidemiology'/de OR 'geographic distribution'/de OR incidence/exp OR 'patient volume'/de OR prevalence/exp OR geography/de OR 'geographic names'/exp OR (epidemiolog* OR ((geograph* OR global*) NEAR/3 (distribut*)) OR incidenc* OR (patient* NEAR/3 volume*) OR prevalen*):ab,ti) NOT ([animals]/lim NOT [humans]/lim) NOT ('case report'/de OR 'case report*':ti) NOT ([Conference Abstract]/lim) AND [english]/lim

Medline Ovid

(Non-alcoholic Fatty Liver Disease/ OR (((nonalcohol* OR non-alcohol*) ADJ3 (fatty-liver* OR steatohepat* OR fld OR hepatosteato*)) OR ((nonalcohol* OR non-alcohol*) ADJ3 steato* ADJ3 (hepat* OR liver*)) OR nafld).ab,ti.) AND (Epidemiological Monitoring/ OR Epidemiology/ OR Epidemiology.fs. OR exp Incidence/ OR exp Prevalence/ OR Geography/ OR exp Geographic Locations/ OR (epidemiolog* OR ((geograph* OR global*) ADJ3 (distribut*)) OR incidenc* OR (patient* ADJ3 volume*) OR prevalen*).ab,ti.) NOT (exp animals/ NOT humans/) NOT (case report/ OR case report*.ti.) NOT (news OR congres* OR abstract* OR book* OR chapter* OR dissertation abstract*).pt. AND english.la.

Web of science Core Collection

TS=(((((nonalcohol* OR non-alcohol*) NEAR/2 (fatty-liver* OR steatohepat* OR fld OR hepatosteato*)) OR ((nonalcohol* OR non-alcohol*) NEAR/2 steato* NEAR/2 (hepat* OR liver*)) OR nafld)) AND ((epidemiolog* OR ((geograph* OR global*) NEAR/2 (distribut*)) OR incidenc* OR (patient* NEAR/2 volume*) OR prevalen*)) NOT ((animal* OR rat OR rats OR mouse OR mice OR murine OR dog OR dogs OR canine OR cat OR cats OR feline OR rabbit OR cow OR cows OR bovine OR rodent* OR sheep OR ovine OR pig OR swine OR porcine OR veterinar* OR chick* OR zebrafish* OR baboon* OR nonhuman* OR primate* OR cattle* OR goose OR geese OR duck OR macaque* OR avian* OR bird* OR fish*) NOT (human* OR patient* OR women OR woman OR men OR man))) NOT TI=( "case report*") AND LA=(english) AND DT=(article) Cochrane CENTRAL ((((nonalcohol* OR non-alcohol*) NEAR/3 (fatty-liver* OR steatohepat* OR fld OR hepatosteato*)) OR ((nonalcohol* OR non-alcohol*) NEAR/3 steato* NEAR/3 (hepat* OR liver*)) OR nafld):ab,ti) AND ((epidemiolog* OR ((geograph* OR global*) NEAR/3 (distribut*)) OR incidenc* OR (patient* NEAR/3 volume*) OR prevalen*):ab,ti)

**Supplementary table 1. Characteristics for included studies.**

| **Study** | **Country/ Region** | **Publication Year** | **Study Time** | **Study Design** | **Sample Source** | **Diagnostic Technique** | **MAFLD** | **Individuals** | **Body Mass Index** | **Study source** | **Quality** |
| --- | --- | --- | --- | --- | --- | --- | --- | --- | --- | --- | --- |
| **General population** | | | | | | | | | | | |
| Adibi.A^1^ | Iran | 2009 | 2006-2007 | Cross-sectional | Community | US | 157 | 544 | BMI >85th percentile with age- and sex-adjustment | Fatty liver | 8 |
| Alavian.SM^2^ | Iran | 2008 | 2007 | Cross-sectional | Community | US | 45 | 267 | BMI>25 | NAFLD | 8 |
| Parray.IA^3^ | India | 2013 | 2008-2010 | Cross-sectional | Community | US | 23 | 42 | Not specified | NAFLD | 8 |
| Pawar.SV^4^ | India | 2016 | 2016 | Cross-sectional | Community | US | 123 | 198 | Overweight and obese was done using both Khadilkar criteria and Cole criteria | NAFLD | 7 |
| Geurtsen.M^5^ | Netherlands | 2019 | 2002-2006 | Cross-sectional | Hospital | MRI | 64 | 548 | BMI >85th percentile | NAFLD | 8 |
| Kazemi.SA^6^ | Iran | 2016 | 2016 | Cross-sectional | Community | US | 104 | 145 | BMI >85th percentile | Fatty liver | 7 |
| Fu.CC^7^ | Taiwan | 2009 | 2002 | Cross-sectional | Community | US | 71 | 122 | BMI >85th percentile | NAFLD | 8 |
| Khalkhali.HR^8^ | Iran | 2016 | 2013 | Cross-sectional | School | US | 132 | 150 | BMI >85th percentile | Fatty liver | 7 |
| Lin.M^9^ | Taiwan | 2017 | 2012-2013 | Cross-sectional | Community | US | 35 | 197 | BMI >95th percentile | NAFLD | 7 |
| Lin.YC^10^ | Taiwan | 2012 | 2006-2011 | Cross-sectional | Community | US | 182 | 781 | BMI >95th percentile | NAFLD | 8 |
| Tominaga.K^11^ | Japan | 1995 | 1989 | Cross-sectional | School | US | 20 | 138 | BMI>18 | Fatty liver | 7 |
| Monteiro.PA^12^ | Brazil | 2014 | 2009 | Cross-sectional | Community | US | 45 | 145 | According to the Cole criteria | NAFLD | 8 |
| Namakin.K^13^ | Iran | 2018 | 2018 | Cross-sectional | Community | US | 108 | 202 | BMI >85th percentile | NAFLD | 7 |
| Nier.A^14^ | Germany | 2018 | 2009-2010 | Cross-sectional | School | US | 16 | 89 | Not specified | NAFLD | 8 |
| Ramzan.M^15^ | Pakistan | 2009 | 2009 | Cross-sectional | School | US | 7 | 67 | BMI >85th percentile | Fatty liver | 7 |
| Fernandes.M^16^ | Brazil | 2010 | 2010 | Cross-sectional | Community | US | 14 | 90 | BMI >95th percentile | NAFLD | 7 |
| Black.LJ^17^ | Australia | 2014 | 2006-2008 | Prospective | School | US | 92 | 225 | BMI >85th percentile | NAFLD | 8 |
| Walker.RW^18^ | USA | 2013 | 2013 | Cross-sectional | Community | MRI | 97 | 223 | BMI>25 | Fatty liver | 8 |
| Zhang.XM^19^ | China | 2015 | 2009-2011 | Cross-sectional | Community | US | 241 | 569 | BMI >95th percentile | NAFLD | 7 |
| Tsuruta.G^20^ | Japan | 2010 | 2004-2007 | Cross-sectional | School | US | 21 | 98 | BMI >85th percentile | NAFLD | 8 |
| Zhao.YZ^21^ | China | 2019 | 2017-2018 | Cross-sectional | School | MRI | 40 | 86 | BMI >85th percentile | Fatty liver | 8 |
| Dai.DL^22^ | China | 2017 | 2017 | Cross-sectional | School | US | 39 | 100 | BMI >95th percentile | NAFLD | 8 |
| Huang.SC^23^ | Taiwan | 2013 | 2009 | Cross-sectional | Community | US | 42 | 203 | BMI >85th percentile | NAFLD | 7 |
| Kim.IK^24^ | South Korea | 2008 | 2005 | Cross-sectional | Community | US | 9 | 40 | BMI>25 | Fatty liver | 7 |
| Lin.YC^25^ | Taiwan | 2009 | 2006 | Cross-sectional | Community | US | 28 | 234 | BMI >95th percentile | NAFLD | 8 |
| Monteiro.PA^26^ | Brazil | 2013 | 2010 | Cross-sectional | Community | US | 48 | 190 | BMI >95th percentile | NAFLD | 8 |
| Berna.SJD^27^ | Spain | 2017 | 2013-2014 | Cross-sectional | School | US | 50 | 148 | BMI z-score > 1 | Fatty liver | 7 |
| Shashaj.B^28^ | Italy | 2014 | 2011-2012 | Cross-sectional | Community | US | 68 | 219 | BMI changed from normal weight to overweight or obesity in the previous 12 months according to the International Obesity Task Force | Fatty liver | 8 |
| Kao.JT^29^ | Taiwan | 2008 | 1999-2005 | Cross-sectional | School | US | 30 | 35 | CDC’s BMI-for-age growth charts for girls and boys in the USA | Fatty liver | 8 |
| **Special population** | | | | | | | | | | | |
| Akcam.M^30^ | Turkey | 2013 | 2010-2011 | Cross-sectional | Hospital | US | 157 | 544 | BMI >95th percentile | NAFLD | 8 |
| Alp.H^31^ | Turkey | 2013 | 2006 | Cross-sectional | Hospital | US | 45 | 267 | BMI >95th percentile | NAFLD | 6 |
| Arenaza.L^32^ | Spain | 2019 | 2017 | Cross-sectional | Hospital | US | 23 | 42 | overweight or obesity according to World Obesity Federation criteria | NAFLD | 8 |
| Arslan.N^33^ | Turkey | 2005 | 2005 | Cross-sectional | Hospital | US | 123 | 198 | BMI of age- and sex matched children in the 50th percentile, then multiplied by 100. The ratio above 120 as obesity | Fatty liver | 6 |
| Boyraz.M^34^ | Turkey | 2013 | 2008-2012 | Cross-sectional | Hospital | MRI | 64 | 548 | BMI >95th percentile | NAFLD | 7 |
| Fu.JF^35^ | China | 2011 | 2004-2009 | Cross-sectional | Hospital | US | 104 | 145 | BMI >95th percentile | NAFLD | 8 |
| Atabek.ME^36^ | Turkey | 2014 | 2014 | Cross-sectional | Hospital | US | 71 | 122 | BMI >95th percentile | NAFLD | 8 |
| Bedogni.G^37^ | Italy | 2012 | 2007-2009 | Cross-sectional | Hospital | US | 132 | 150 | BMI >85th percentile | NAFLD | 8 |
| Belei.O^38^ | Romania | 2017 | 2015-2017 | Cross-sectional | Hospital | US | 35 | 197 | BMI >95th percentile | NAFLD | 7 |
| Benetolo.P^39^ | Brazil | 2018 | 2014-2016 | Cross-sectional | Hospital | US | 182 | 781 | BMI >95th percentile | NAFLD | 7 |
| Burgert.TS^40^ | USA | 2006 | 2004 | Cross-sectional | Hospital | US | 20 | 138 | BMI>18 | NAFLD | 7 |
| Cardoso.AS^41^ | Brazil | 2013 | 2009-2010 | Cross-sectional | Hospital | US | 45 | 145 | BMI>Z score+2 | NAFLD | 7 |
| Chan.DFY^42^ | Hong Kong | 2004 | 2000-2002 | Cross-sectional | Hospital | US | 108 | 202 | BMI >85th percentile | Fatty liver | 7 |
| Daar.G^43^ | Turkey | 2015 | 2013-2014 | Cross-sectional | Hospital | US | 16 | 89 | Two standard deviations above the mean BMI for age and sex | Fatty liver | 7 |
| Das.MK^44^ | India | 2017 | 2017 | Cross-sectional | Hospital | US | 7 | 67 | BMI>18.5 | NAFLD | 8 |
| Zusi.C^45^ | Italy | 2019 | 2019 | Cross-sectional | Hospital | US | 14 | 90 | BMI >95th percentile | NAFLD | 8 |
| Costanzo.DI^46^ | Italy | 2019 | 2019 | Cross-sectional | Hospital | US | 92 | 225 | BMI >85th percentile | NAFLD | 8 |
| Dursun.F^47^ | Turkey | 2019 | 2019 | Cross-sectional | Hospital | MRI | 97 | 223 | BMI>25 | Fatty liver | 8 |
| Karaksy.HM^48^ | Egypt | 2011 | 2011 | Cross-sectional | Hospital | US | 241 | 569 | BMI >95th percentile | NAFLD | 7 |
| Eminoglu.O^49^ | Turkey | 2008 | 2003-2005 | Cross-sectional | Hospital | US | 21 | 98 | BMI >85th percentile | NAFLD | 7 |
| Erol.M^50^ | Turkey | 2016 | 2015 | Cross-sectional | Hospital | MRI | 40 | 86 | BMI >85th percentile | NAFLD | 7 |
| Felix.DR^51^ | Brazil | 2016 | 2010-2013 | Cross-sectional | Hospital | US | 39 | 100 | BMI >95th percentile | NAFLD | 7 |
| Ferraioli.G^52^ | Italy | 2017 | 2012-2016 | Cross-sectional | Hospital | US | 42 | 203 | BMI >85th percentile | NAFLD | 8 |
| Fonvig.CE^53^ | Denmark | 2015 | 2009-2014 | Cross-sectional | Hospital | US | 9 | 40 | BMI>25 | NAFLD | 8 |
| Gheibi.S^54^ | Iran | 2019 | 2016-2017 | Cross-sectional | Hospital | US | 28 | 234 | BMI >95th percentile | NAFLD | 8 |
| Hamza.RT^55^ | Egypt | 2016 | 2011-2013 | Cross-sectional | Hospital | US | 48 | 190 | BMI >95th percentile | NAFLD | 7 |
| Han.XC^56^ | China | 2020 | 2020 | Cross-sectional | Hospital | US | 50 | 148 | BMI z-score > 1 | NAFLD | 8 |
| Hatipoglu.N^57^ | Turkey | 2016 | 2016 | Cross-sectional | Hospital | US | 68 | 219 | BMI changed from normal weight to overweight or obesity in the previous 12 months according to the International Obesity Task Force | NAFLD | 7 |
| Hu.W^58^ | China | 2017 | 2012-2013 | Cross-sectional | Hospital | US | 84 | 169 | BMI >95th percentile | NAFLD | 8 |
| Rivera.CJ^59^ | Canada | 2017 | 2009-2012 | Cross-sectional | Hospital | US | 93 | 400 | BMI >95th percentile | NAFLD | 8 |
| Kalthenbach.TE^60^ | Germany | 2016 | 2000-2001 | Cross-sectional | Hospital | MRI | 40 | 110 | BMI–standard deviation scores (BMI-SDS) based on German reference values. | NAFLD | 8 |
| Wiegand.S^61^ | Germany, Austria and Switzerland | 2010 | 2001-2009 | Cross-sectional | Hospital | US | 38 | 322 | BMI >90th percentile | NAFLD | 8 |
| Zhang.HX^62^ | China | 2014 | 2009-2011 | Cross-sectional | Hospital | US | 217 | 451 | Not specified | NAFLD | 8 |
| Kim.JY^63^ | South Korea | 2018 | 2004-2016 | Cross-sectional | Hospital | US | 587 | 861 | BMI Z score >1 | NAFLD | 8 |
| Kistler.KD^64^ | USA | 2010 | 2007 | Cross-sectional | Hospital | US | 38 | 217 | BMI >85th percentile | NAFLD | 8 |
| Kodhelaj.K^65^ | Albania | 2014 | 2010 | Cross-sectional | School | US | 234 | 571 | BMI >85th percentile | NAFLD | 7 |
| Labayen.I^66^ | Spain | 2018 | 2018 | Cross-sectional | Hospital | US | 36 | 125 | BMI >85th percentile | NAFLD | 8 |
| Lee.JH^67^ | South Korea | 2017 | 2013 | Cross-sectional | Hospital | MRI | 14 | 50 | BMI >85th percentile | NAFLD | 8 |
| Liang.S^68^ | China | 2017 | 2013-2015 | Cross-sectional | Hospital | MRI | 125 | 392 | BMI >95th percentile | NAFLD | 8 |
| Mameli.C^69^ | Italy | 2018 | 2010-2017 | Cross-sectional | Hospital | US | 37 | 129 | BMI Z score >1 | Fatty liver | 8 |
| De piano.A^70^ | Brazil | 2007 | 2007 | Cross-sectional | Hospital | US | 65 | 84 | BMI>30 | NAFLD | 7 |
| Mohammed.RZ^71^ | Malaysia | 2020 | 2015 | Cross-sectional | Hospital | US | 43 | 53 | BMI >85th percentile | NAFLD | 7 |
| Ardakali.AT^72^ | Iran | 2014 | 2012 | Cross-sectional | Hospital | US | 215 | 961 | BMI>18.5 | Fatty liver | 7 |
| Navarro.JM^73^ | Spain | 2013 | 2013 | Cross-sectional | Hospital | US | 347 | 514 | BMI >95th percentile | NAFLD | 8 |
| Franzese.A^74^ | Italy | 1997 | 1997 | Cross-sectional | Hospital | MRI | 105 | 230 | BMI >85th percentile | Fatty liver | 8 |
| Guzzaloni.G^75^ | Italy | 2000 | 2000 | Cross-sectional | Hospital | US | 49 | 110 | BMI >95th percentile | Fatty liver | 8 |
| Oh.MS^76^ | South Korea | 2019 | 2015 | Cross-sectional | Hospital | Biopsy | 33 | 76 | BMI >85th percentile | NAFLD | 7 |
| Ozhan.B^77^ | Turkey | 2016 | 2016 | Cross-sectional | Hospital | US | 53 | 101 | BMI >95th percentile | NAFLD | 7 |
| Pacifico.L^78^ | Italy | 2016 | 2007-2015 | Cross-sectional | Hospital | US | 62 | 107 | BMI >85th percentile | NAFLD | 8 |
| Papandreou.D^79^ | Greece | 2012 | 2007-2011 | Cross-sectional | Hospital | US | 8 | 39 | BMI >95th percentile | NAFLD | 7 |
| Pozzato.C^80^ | Italy | 2008 | 2006-2007 | Cross-sectional | Hospital | FLI | 44 | 199 | According to the Cole criteria | NAFLD | 7 |
| Sagi.R^81^ | Israel | 2007 | 2003-2004 | Cross-sectional | Hospital | MRI | 89 | 287 | BMI >90th percentile | NAFLD | 8 |
| Prokopowicz.Z^82^ | Poland | 2018 | 2012-2014 | Cross-sectional | Hospital | US | 69 | 747 | LMS method based on Polish reference values | NAFLD | 8 |
| Radetti.G^83^ | Italy | 2006 | 2004 | Cross-sectional | Hospital | US | 40 | 50 | BMI >95th percentile | NAFLD | 7 |
| Ruiz-Extremera.A^84^ | Spain | 2011 | 2011 | Cross-sectional | Hospital | US | 262 | 620 | BMI >95th percentile | Fatty liver | 7 |
| Schlieske.C^85^ | Germany | 2014 | 2014 | Cross-sectional | Hospital | US | 88 | 248 | BMI >95th percentile | NAFLD | 7 |
| Damaso.AR^86^ | Brazil | 2008 | 2008 | Cross-sectional | Hospital | US | 40 | 71 | BMI >95th percentile | NAFLD | 7 |
| Jung.JH^87^ | South Korea | 2015 | 2013-2014 | Retrospective | Hospital | US | 82 | 97 | BMI >95th percentile | NAFLD | 8 |
| Sezer.OB^88^ | Turkey | 2016 | 2015 | Cross-sectional | Hospital | US | 99 | 332 | BMI >95th percentile | Fatty liver | 7 |
| Yildiz.I^89^ | Turkey | 2014 | 2012-2013 | Cross-sectional | Hospital | US | 880 | 10204 | BMI >90th percentile | Fatty liver | 7 |
| Pirgon.O^90^ | Turkey | 2013 | 2010-2011 | Cross-sectional | Hospital | H-MRS | 111 | 171 | BMI >95th percentile | NAFLD | 7 |
| Cali.AMG^91^ | USA | 2007 | 2007 | Cross-sectional | Hospital | US | 192 | 352 | BMI >90th percentile | NAFLD | 6 |
| Shi.JQ^92^ | China | 2017 | 2015 | Cross-sectional | Hospital | Biopsy | 148 | 239 | BMI >85th percentile | NAFLD | 8 |
| Torun.E^93^ | Turkey | 2014 | 2011-2013 | Cross-sectional | Hospital | US | 55 | 80 | BMI >85th percentile | NAFLD | 7 |
| Ustyol.A^94^ | Turkey | 2017 | 2015-2016 | Cross-sectional | Hospital | MRI | 41 | 115 | Not specified | NAFLD | 8 |
| Vakilishahrbabaki.HS^95^ | Iran | 2018 | 2016-2017 | case-control | Hospital | US | 144 | 242 | BMI >85th percentile | NAFLD | 7 |
| Trico.D^96^ | USA | 2018 | 2018 | Cross-sectional | Hospital | US | 90 | 168 | BMI >95th percentile | NAFLD | 8 |
| Wang.R^97^ | China | 2018 | 2015-2016 | Cross-sectional | Hospital | US | 46 | 213 | BMI z score >1 | NAFLD | 8 |
| Yang.HR^98^ | South Korea | 2016 | 2012-2014 | Cross-sectional | Hospital | US | 13 | 43 | BMI>30 | NAFLD | 8 |
| Silveira.LS^99^ | Brazil | 2013 | 2013 | Cross-sectional | Hospital | US | 21 | 33 | BMI >85th percentile | NAFLD | 8 |
| Wasilewska.N^100^ | Poland | 2018 | 2018 | Cross-sectional | Hospital | US | 120 | 200 | BMI >95th percentile | NAFLD | 8 |
| Yang.HR^101^ | South Korea | 2014 | 2006-2012 | Cross-sectional | Hospital | US | 50 | 144 | BMI >95th percentile | NAFLD | 8 |
| Grønbæk.H^102^ | Denmark | 2012 | 2011 | Cross-sectional | Hospital | US | 38 | 72 | An ideal body weight higher than 160% was considered as morbid obesity. All patients had an IBW over 120%. | NAFLD | 8 |
| Saad.V^103^ | Canada | 2015 | 2008-2012 | Cross-sectional | Hospital | US | 157 | 375 | BMI > 2 s.d. for chronological age | NAFLD | 7 |
| Zou.CC^104^ | China | 2005 | 2005 | Cross-sectional | Hospital | US | 41 | 212 | BMI >85th percentile | NAFLD | 6 |
| Bohte.AE^105^ | Netherlands | 2011 | 2008-2010 | Cross-sectional | Hospital | US | 202 | 332 | BMI >95th percentile | Fatty liver | 7 |
| Chiloiro.M^106^ | Italy | 2008 | 2008 | Cross-sectional | Hospital | US | 268 | 596 | BMI Z-score >2 | Fatty liver | 6 |
| Silva.KSH^107^ | Sri Lanka | 2006 | 2004-2005 | Cross-sectional | Hospital | US | 38 | 85 | BMI >95th percentile | NAFLD | 7 |
| Denzer.C^108^ | Germany | 2009 | 2009 | Cross-sectional | Hospital | MRI | 14 | 60 | BMI > 2 s.d. for chronological age | NAFLD | 8 |
| Hacihamdioglu.B^109^ | Turkey | 2011 | 2011 | Cross-sectional | Hospital | US | 35 | 58 | BMI >85th percentile | NAFLD | 6 |
| Kim.JS^110^ | USA | 2012 | 2012 | Cross-sectional | Hospital | US | 34 | 108 | BMI >97th percentile | NAFLD | 6 |
| El-Koofy.NM^111^ | Egypt | 2012 | 2012 | Cross-sectional | Hospital | MRI | 14 | 44 | BMI >97th percentile | NAFLD | 9 |
| Ozkol.M^112^ | Turkey | 2010 | 2010 | Cross-sectional | Hospital | US | 57 | 127 | BMI >95th percentile | Fatty liver | 7 |
| Papandreou.D^113^ | Greece | 2008 | 2005-2006 | Cross-sectional | Hospital | US | 121 | 447 | BMI >90th percentile | Fatty liver | 6 |
| Perseghin.G^114^ | Italy | 2006 | 2006 | Cross-sectional | Hospital | US | 82 | 181 | BMI >95th percentile | NAFLD | 7 |
| Reinehr.T^115^ | Germany | 2008 | 2008 | Cross-sectional | Hospital | US | 28 | 64 | BMI >85th percentile | NAFLD | 7 |
| Tock.L^116^ | Brazil | 2006 | 2006 | Cross-sectional | Hospital | US | 58 | 113 | BMI >95th percentile | NAFLD | 7 |
| Xanthakos.S^117^ | USA | 2006 | 2003-2005 | Cross-sectional | Hospital | US | 58 | 101 | BMI >95th percentile | NAFLD | 8 |
| Boza.C^118^ | Chile | 2011 | 2006-2009 | Cross-sectional | Hospital | US | 45 | 87 | BMI >95th percentile | NAFLD | 9 |
| Koot.BG^119^ | Netherlands | 2011 | 2004-2008 | Cross-sectional | Hospital | MRI | 12 | 49 | BMI >95th percentile | NAFLD | 8 |
| Alqahtani.A^120^ | Saudi Arabia | 2017 | 2008 | Cross-sectional | Hospital | US | 43 | 117 | BMI >95th percentile | NAFLD | 9 |
| Chociej.AB^121^ | Spain | 2018 | 2018 | Cross-sectional | Hospital | US | 85 | 109 | BMI >95th percentile | NAFLD | 8 |
| Deeb.A^122^ | United Arab Emirates | 2018 | 2018 | Cross-sectional | Hospital | US | 80 | 228 | BMI >95th percentile | Fatty liver | 8 |
| Assuncao.SNF^123^ | Brazil | 2017 | 2015 | Cross-sectional | Hospital | US | 126 | 358 | BMI >95th percentile | NAFLD | 7 |
| Goyal.P^124^ | India | 2018 | 2018 | Cross-sectional | Hospital | MRI | 209 | 503 | BMI >95th percentile | NAFLD | 8 |
| Kirel.B^125^ | Turkey | 2012 | 2007-2010 | Cross-sectional | Hospital | US | 58 | 123 | BMI >95th percentile | NAFLD | 7 |
| Martino.MD^126^ | Italy | 2016 | 2013-2014 | Cross-sectional | Hospital | US | 48 | 182 | BMI >95th percentile | NAFLD | 7 |
| Yu.EL^127^ | USA | 2018 | 2018 | Cross-sectional | Hospital | US | 31 | 80 | BMI >95th percentile | NAFLD | 7 |
| Szyboska.P^128^ | Poland | 2015 | 2015 | Cross-sectional | Hospital | US | 147 | 396 | BMI >85th percentile | NAFLD | 7 |
| Pena-Velez.R^129^ | Mexico | 2019 | 2019 | Cross-sectional | Hospital | US | 49 | 117 | BMI >95th percentile | NAFLD | 8 |
| Elkabany.ZA^130^ | Egypt | 2019 | 2019 | Cross-sectional | Hospital | MRI | 42 | 129 | BMI >85th percentile | NAFLD | 7 |
| Ozsu.E^131^ | Turkey | 2019 | 2014-2015 | Cross-sectional | Hospital | US | 63 | 113 | Body weight exceeded 120% standard body weight | NAFLD | 7 |
| Hua.MC^132^ | Taiwan | 2019 | 2015-2017 | Cross-sectional | Hospital | US | 48 | 104 | BMI z score of greater than 2 (95th percentile | NAFLD | 8 |
| Bonito.PD^133^ | Italy | 2019 | 2003-2016 | Cross-sectional | Hospital | US | 60 | 94 | BMI >95th percentile | NAFLD | 7 |
| Jain.V^134^ | India | 2019 | 2012-2016 | Cross-sectional | Hospital | US | 60 | 70 | BMI >95th percentile | NAFLD | 7 |
| Ortega.EV^135^ | Mexico | 2019 | 2015-2016 | Cross-sectional | Hospital | US | 149 | 532 | BMI >90th percentile | NAFLD | 7 |
| Draijer.LG^136^ | Netherlands | 2019 | 2008-2010 | Cross-sectional | Hospital | US | 21 | 104 | BMI >95th percentile | NAFLD | 7 |
| Kurku.H^137^ | Turkey | 2019 | 2016-2017 | Cross-sectional | Hospital | MRI | 16 | 41 | BMI >85th percentile | NAFLD | 7 |
| Velez.RR^138^ | Colombia | 2018 | 2018 | Cross-sectional | Hospital | Biopsy | 15 | 33 | BMI >85th percentile | NAFLD | 7 |
| Jackiewicz.MF^139^ | Poland | 2018 | 2018 | Cross-sectional | Hospital | US | 30 | 59 | BMI >85th percentile | NAFLD | 8 |
| Bacha.F^140^ | USA | 2017 | 2017 | Cross-sectional | Hospital | US | 18 | 43 | BMI >95th percentile | NAFLD | 7 |
| Balanescu.A^141^ | Romania | 2018 | 2017 | Cross-sectional | Hospital | H-MRS | 16 | 54 | BMI >99th percentile | NAFLD | 7 |
| Lu.LP^142^ | China | 2017 | 2013-2015 | Cross-sectional | Hospital | US | 12 | 36 | BMI >95th percentile | NAFLD | 7 |
| Chabanova.E^143^ | Denmark | 2017 | 2017 | Cross-sectional | Hospital | US | 38 | 73 | BMI >95th percentile | NAFLD | 8 |
| Lee.SJ^144^ | USA | 2016 | 2016 | Cross-sectional | Hospital | Biopsy | 34 | 41 | BMI >99th percentile | NAFLD | 7 |
| Sheldon.RD^145^ | Colombia | 2016 | 2016 | Cross-sectional | Hospital | Biopsy | 8 | 51 | BMI 40 mg/kg^2^ or BMI 35 mg/kg^2^ with associated co-morbidities | NAFLD | 9 |
| Jin.R^146^ | USA | 2015 | 2015 | Cross-sectional | Hospital | US | 45 | 144 | BMI>35 or BMI>30 with comorbidity | NAFLD | 7 |
| Chang.PF^147^ | Taiwan | 2015 | 2010-2012 | Cross-sectional | Hospital | Biopsy | 114 | 144 | body mass index (BMI) of 40 kg/m^2^ (or multiple co-morbidities with a BMI 43.5 kg/m^2^ or above the 99th percentile for age), | NAFLD | 8 |
| Oksiuta.MK^148^ | Japan | 2014 | 2011-2012 | Cross-sectional | Hospital | US | 38 | 56 | BMI >95th percentile | NAFLD | 7 |
| O'Sullivan.TA^149^ | Austrilia | 2013 | 2003-2005 | Prospective | Hospital | US | 43 | 121 | BMI >85th percentile | NAFLD | 8 |
| Santoro.N^150^ | USA | 2014 | 2014 | Cross-sectional | Hospital | US | 56 | 100 | BMI>+2 | NAFLD | 7 |
| [Lebensztejn.DM](https://pubmed.ncbi.nlm.nih.gov/?sort=date&term=Lebensztejn+DM&cauthor_id=20371430)^151^ | Poland | 2010 | 2006-2007 | Cross-sectional | Hospital | US | 106 | 160 | BMI>26 | NAFLD | 6 |
| Demircioglu.F^152^ | Turkey | 2008 | 2004 | Case-control | Hospital | US | 66 | 161 | BMI >95th percentile | NAFLD | 7 |
| Osborne.KNL^153^ | USA | 2008 | 2008 | Cross-sectional | Hospital | MRI | 40 | 65 | BMI>30 or BMI >95th percentile | NAFLD | 7 |
| Shalitin.S^154^ | Israel | 2008 | 2004-2006 | Cross-sectional | Hospital | Biopsy | 27 | 93 | BMI >95th percentile | Fatty liver | 8 |
| Nichols.PH^155^ | USA | 2019 | 2010-2018 | Cross-sectional | Hospital | MRI | 106 | 408 | BMI >85th percentile | NAFLD | 7 |
| Barretto.JR^156^ | Brazil | 2020 | 2019 | Cross-sectional | Hospital | US | 20 | 58 | BMI Z-score >1 | NAFLD | 7 |

*US, ultrasound; CT, computed tomography; MRI, magnetic resonance imaging; FLI, fatty liver index; H-MRS, proton magnetic resonance spectroscopy; BMI, body mass index.

**Supplementary Table 2. Leave-one-out analysis for MAFLD prevalence in general population.**

|  | **estimate** | **zval** | **pval** | **ci.lb** | **ci.ub** | **Q** | **Qp** | **tau2** | **I2** | **H2** |
| --- | --- | --- | --- | --- | --- | --- | --- | --- | --- | --- |
| 1 | 0.3399 | -4.0071 | 0.0001 | 0.2712 | 0.416 | 712.3002 | 0 | 0.7186 | 96.2095 | 26.3815 |
| 2 | 0.3454 | -4.0593 | 0 | 0.2793 | 0.4181 | 686.4295 | 0 | 0.6451 | 96.0666 | 25.4233 |
| 3 | 0.3314 | -4.4527 | 0 | 0.2668 | 0.403 | 708.5794 | 0 | 0.6492 | 96.1896 | 26.2437 |
| 4 | 0.328 | -4.6671 | 0 | 0.2654 | 0.3975 | 646.9158 | 0 | 0.6118 | 95.8263 | 23.9598 |
| 5 | 0.3491 | -4.1409 | 0 | 0.2853 | 0.4187 | 613.899 | 0 | 0.5848 | 95.6019 | 22.737 |
| 6 | 0.3247 | -4.8452 | 0 | 0.2633 | 0.3927 | 636.092 | 0 | 0.5912 | 95.7553 | 23.559 |
| 7 | 0.3296 | -4.5328 | 0 | 0.2656 | 0.4006 | 684.235 | 0 | 0.6381 | 96.054 | 25.342 |
| 8 | 0.3171 | -5.2567 | 0 | 0.2586 | 0.382 | 600.0112 | 0 | 0.5488 | 95.5001 | 22.2226 |
| 9 | 0.3448 | -4.0634 | 0 | 0.2786 | 0.4177 | 696.9079 | 0 | 0.6498 | 96.1257 | 25.8114 |
| 10 | 0.3423 | -3.9876 | 0.0001 | 0.2741 | 0.4177 | 679.6332 | 0 | 0.701 | 96.0273 | 25.1716 |
| 11 | 0.3465 | -4.04 | 0.0001 | 0.2804 | 0.419 | 697.2219 | 0 | 0.6427 | 96.1275 | 25.823 |
| 12 | 0.3388 | -4.1694 | 0 | 0.2724 | 0.4124 | 716.7716 | 0 | 0.6706 | 96.2331 | 26.5471 |
| 13 | 0.331 | -4.4633 | 0 | 0.2665 | 0.4026 | 679.4483 | 0 | 0.6462 | 96.0262 | 25.1648 |
| 14 | 0.3443 | -4.0797 | 0 | 0.2781 | 0.4171 | 708.3022 | 0 | 0.6507 | 96.1881 | 26.2334 |
| 15 | 0.3479 | -4.0096 | 0.0001 | 0.2819 | 0.4204 | 703.9366 | 0 | 0.6422 | 96.1644 | 26.0717 |
| 16 | 0.3455 | -4.0561 | 0 | 0.2794 | 0.4182 | 705.4478 | 0 | 0.6472 | 96.1726 | 26.1277 |
| 17 | 0.3353 | -4.2399 | 0 | 0.2688 | 0.409 | 710.5131 | 0 | 0.6797 | 96.1999 | 26.3153 |
| 18 | 0.3344 | -4.2785 | 0 | 0.2682 | 0.4078 | 705.6339 | 0 | 0.6751 | 96.1737 | 26.1346 |
| 19 | 0.3348 | -4.1557 | 0 | 0.2669 | 0.4103 | 691.9041 | 0 | 0.7137 | 96.0977 | 25.6261 |
| 20 | 0.3427 | -4.1078 | 0 | 0.2765 | 0.4157 | 711.1592 | 0 | 0.6556 | 96.2034 | 26.3392 |
| 21 | 0.3335 | -4.3587 | 0 | 0.2683 | 0.4059 | 710.0343 | 0 | 0.6577 | 96.1974 | 26.2976 |
| 22 | 0.336 | -4.2684 | 0 | 0.2701 | 0.4089 | 715.4075 | 0 | 0.6645 | 96.2259 | 26.4966 |
| 23 | 0.3433 | -4.0787 | 0 | 0.2769 | 0.4166 | 703.1083 | 0 | 0.6587 | 96.1599 | 26.041 |
| 24 | 0.3417 | -4.15 | 0 | 0.2759 | 0.4144 | 715.0685 | 0 | 0.6532 | 96.2241 | 26.484 |
| 25 | 0.3485 | -4.0302 | 0.0001 | 0.2829 | 0.4203 | 675.1673 | 0 | 0.6262 | 96.001 | 25.0062 |
| 26 | 0.3412 | -4.1081 | 0 | 0.2745 | 0.4148 | 711.7465 | 0 | 0.6688 | 96.2065 | 26.361 |
| 27 | 0.3378 | -4.1931 | 0 | 0.2714 | 0.4113 | 717.0026 | 0 | 0.6721 | 96.2343 | 26.5557 |
| 28 | 0.3389 | -4.1378 | 0 | 0.2719 | 0.413 | 716.6345 | 0 | 0.6808 | 96.2324 | 26.542 |
| 29 | 0.3226 | -4.7931 | 0 | 0.2601 | 0.3921 | 690.189 | 0 | 0.6279 | 96.088 | 25.5626 |

**Supplementary Table 3. Leave-one-out diagnostics with a built-in function in MAFLD prevalence in general population.**

|  | **rstudent** | **dffits** | **cook.d** | **cov.r** | **tau2.del** | **QE.del** | **hat** | **weight** | **dfbs** | **inf** |
| --- | --- | --- | --- | --- | --- | --- | --- | --- | --- | --- |
| 1 | -0.2742 | -0.0568 | 0.0036 | 1.1407 | 0.7186 | 712.3002 | 0.0365 | 3.6549 | -0.057 |  |
| 2 | -1.1464 | -0.22 | 0.0481 | 1.0302 | 0.6451 | 686.4295 | 0.0356 | 3.5588 | -0.22 |  |
| 3 | 1.0176 | 0.1859 | 0.0345 | 1.0329 | 0.6492 | 708.5794 | 0.0323 | 3.2277 | 0.1859 |  |
| 4 | 1.4952 | 0.2914 | 0.0802 | 0.981 | 0.6118 | 646.9158 | 0.0359 | 3.5867 | 0.2911 |  |
| 5 | -1.7707 | -0.3396 | 0.1042 | 0.9409 | 0.5848 | 613.899 | 0.0361 | 3.607 | -0.3389 |  |
| 6 | 2.0661 | 0.3992 | 0.1458 | 0.9495 | 0.5912 | 636.092 | 0.0352 | 3.5209 | 0.3989 |  |
| 7 | 1.2473 | 0.2391 | 0.0562 | 1.0194 | 0.6381 | 684.235 | 0.0352 | 3.5225 | 0.2391 |  |
| 8 | 3.4679 | 0.6528 | 0.3659 | 0.8847 | 0.5488 | 600.0112 | 0.0338 | 3.377 | 0.6542 | * |
| 9 | -1.057 | -0.2018 | 0.0407 | 1.0368 | 0.6498 | 696.9079 | 0.0352 | 3.5171 | -0.2018 |  |
| 10 | -0.6277 | -0.1248 | 0.0168 | 1.1146 | 0.701 | 679.6332 | 0.0366 | 3.6648 | -0.125 |  |
| 11 | -1.3386 | -0.2506 | 0.0622 | 1.0249 | 0.6427 | 697.2219 | 0.034 | 3.3992 | -0.2507 |  |
| 12 | -0.1524 | -0.0304 | 0.0009 | 1.0679 | 0.6706 | 716.7716 | 0.0353 | 3.5301 | -0.0304 |  |
| 13 | 1.0134 | 0.1959 | 0.0382 | 1.0323 | 0.6462 | 679.4483 | 0.036 | 3.5951 | 0.1959 |  |
| 14 | -1.0076 | -0.1867 | 0.0349 | 1.036 | 0.6507 | 708.3022 | 0.0332 | 3.3162 | -0.1867 |  |
| 15 | -1.6726 | -0.292 | 0.0845 | 1.0196 | 0.6422 | 703.9366 | 0.0297 | 2.9747 | -0.2922 |  |
| 16 | -1.2106 | -0.2227 | 0.0494 | 1.0303 | 0.6472 | 705.4478 | 0.0328 | 3.2783 | -0.2227 |  |
| 17 | 0.371 | 0.0699 | 0.0051 | 1.0823 | 0.6797 | 710.5131 | 0.036 | 3.6032 | 0.0699 |  |
| 18 | 0.5032 | 0.0957 | 0.0095 | 1.0754 | 0.6751 | 705.6339 | 0.036 | 3.604 | 0.0958 |  |
| 19 | 0.4372 | 0.0814 | 0.0073 | 1.1336 | 0.7137 | 691.9041 | 0.0366 | 3.6646 | 0.0816 |  |
| 20 | -0.7527 | -0.1414 | 0.0202 | 1.0441 | 0.6556 | 711.1592 | 0.0339 | 3.389 | -0.1414 |  |
| 21 | 0.6467 | 0.1219 | 0.015 | 1.048 | 0.6577 | 710.0343 | 0.0346 | 3.4565 | 0.1219 |  |
| 22 | 0.2734 | 0.051 | 0.0027 | 1.0583 | 0.6645 | 715.4075 | 0.0348 | 3.48 | 0.051 |  |
| 23 | -0.8227 | -0.1581 | 0.0253 | 1.0504 | 0.6587 | 703.1083 | 0.0354 | 3.5416 | -0.1582 |  |
| 24 | -0.6413 | -0.1138 | 0.013 | 1.0367 | 0.6532 | 715.0685 | 0.0304 | 3.0353 | -0.1137 |  |
| 25 | -1.6483 | -0.3119 | 0.094 | 1.0013 | 0.6262 | 675.1673 | 0.0349 | 3.4874 | -0.3119 |  |
| 26 | -0.5021 | -0.0974 | 0.0098 | 1.0655 | 0.6688 | 711.7465 | 0.0355 | 3.5528 | -0.0975 |  |
| 27 | 0.0001 | -0.0013 | 0 | 1.0703 | 0.6721 | 717.0026 | 0.0354 | 3.5406 | -0.0013 |  |
| 28 | -0.1517 | -0.031 | 0.001 | 1.0838 | 0.6808 | 716.6345 | 0.0359 | 3.5875 | -0.031 |  |
| 29 | 2.6931 | 0.4491 | 0.1967 | 0.9958 | 0.6279 | 690.189 | 0.0273 | 2.726 | 0.4506 |  |

**Supplementary Table 4. Univariate meta-regression for MAFLD prevalence in general population.**

| **Covariate** | **Results** |
| --- | --- |
| **Study size** | Mixed-Effects Model (k = 29; tau^2 estimator: DL)  tau^2 (estimated amount of residual heterogeneity): 0.0406 (SE = 0.0149)  tau (square root of estimated tau^2 value): 0.2015  I^2 (residual heterogeneity / unaccounted variability): 98.02%  H^2 (unaccounted variability / sampling variability): 50.55  R^2 (amount of heterogeneity accounted for): 0.00%  Test for Residual Heterogeneity:  QE(df = 27) = 1364.7415, p-val < .0001  Test of Moderators (coefficient 2):  F(df1 = 1, df2 = 27) = 0.0728, p-val = 0.7894  Model Results:  estimate se tval pval ci.lb ci.ub  intrcpt 0.3137 0.1658 1.8923 0.0692 -0.0264 0.6538 .  size 0.0250 0.0927 0.2698 0.7894 -0.1652 0.2152  ---  Signif. codes: 0 ‘***’ 0.001 ‘**’ 0.01 ‘*’ 0.05 ‘.’ 0.1 ‘ ’ 1 |
| **Study quality** | Mixed-Effects Model (k = 29; tau^2 estimator: DL)  tau^2 (estimated amount of residual heterogeneity): 0.0357 (SE = 0.0131)  tau (square root of estimated tau^2 value): 0.1890  I^2 (residual heterogeneity / unaccounted variability): 97.69%  H^2 (unaccounted variability / sampling variability): 43.20  R^2 (amount of heterogeneity accounted for): 10.12%  Test for Residual Heterogeneity:  QE(df = 27) = 1166.4384, p-val < .0001  Test of Moderators (coefficient 2):  F(df1 = 1, df2 = 27) = 0.2041, p-val = 0.6551  Model Results:  estimate se tval pval ci.lb ci.ub  intrcpt 0.3037 0.1244 2.4404 0.0215 0.0483 0.5590 *  quality 0.0375 0.0830 0.4518 0.6551 -0.1328 0.2079  ---  Signif. codes: 0 ‘***’ 0.001 ‘**’ 0.01 ‘*’ 0.05 ‘.’ 0.1 ‘ ’ 1 |
| **Publication time** | Mixed-Effects Model (k = 29; tau^2 estimator: DL)  tau^2 (estimated amount of residual heterogeneity): 0.0397 (SE = 0.0145)  tau (square root of estimated tau^2 value): 0.1993  I^2 (residual heterogeneity / unaccounted variability): 97.90%  H^2 (unaccounted variability / sampling variability): 47.66  R^2 (amount of heterogeneity accounted for): 0.11%  Test for Residual Heterogeneity:  QE(df = 27) = 1286.7412, p-val < .0001  Test of Moderators (coefficient 2):  F(df1 = 1, df2 = 27) = 1.8371, p-val = 0.1865  Model Results:  estimate se tval pval ci.lb ci.ub  intrcpt 0.1690 0.1442 1.1718 0.2515 -0.1269 0.4650  public 0.1134 0.0837 1.3554 0.1865 -0.0583 0.2852  ---  Signif. codes: 0 ‘***’ 0.001 ‘**’ 0.01 ‘*’ 0.05 ‘.’ 0.1 ‘ ’ 1 |
| **Country or regional income** | Mixed-Effects Model (k = 29; tau^2 estimator: DL)  tau^2 (estimated amount of residual heterogeneity): 0.0328 (SE = 0.0124)  tau (square root of estimated tau^2 value): 0.1811  I^2 (residual heterogeneity / unaccounted variability): 97.53%  H^2 (unaccounted variability / sampling variability): 40.44  R^2 (amount of heterogeneity accounted for): 17.46%  Test for Residual Heterogeneity:  QE(df = 25) = 1010.8868, p-val < .0001  Test of Moderators (coefficients 2:4):  F(df1 = 3, df2 = 25) = 1.1675, p-val = 0.3419  Model Results:  estimate se tval pval ci.lb ci.ub  intrcpt 0.3001 0.0555 5.4071 <.0001 0.1858 0.4144 ***  incomelower 0.3211 0.2214 1.4501 0.1595 -0.1349 0.7772  incomeLower 0.0123 0.1662 0.0738 0.9418 -0.3300 0.3546  incomeUpper 0.1166 0.0852 1.3678 0.1835 -0.0590 0.2921  ---  Signif. codes: 0 ‘***’ 0.001 ‘**’ 0.01 ‘*’ 0.05 ‘.’ 0.1 ‘ ’ 1 |
| **Country development** | Mixed-Effects Model (k = 29; tau^2 estimator: DL)  tau^2 (estimated amount of residual heterogeneity): 0.0395 (SE = 0.0139)  tau (square root of estimated tau^2 value): 0.1987  I^2 (residual heterogeneity / unaccounted variability): 97.84%  H^2 (unaccounted variability / sampling variability): 46.38  R^2 (amount of heterogeneity accounted for): 0.70%  Test for Residual Heterogeneity:  QE(df = 27) = 1252.2837, p-val < .0001  Test of Moderators (coefficient 2):  F(df1 = 1, df2 = 27) = 2.5364, p-val = 0.1229  Model Results:  estimate se tval pval ci.lb ci.ub  intrcpt 0.2637 0.0705 3.7403 0.0009 0.1190 0.4083 ***  deveing 0.1352 0.0849 1.5926 0.1229 -0.0390 0.3094  ---  Signif. codes: 0 ‘***’ 0.001 ‘**’ 0.01 ‘*’ 0.05 ‘.’ 0.1 ‘ ’ 1 |
| **Diagnostic technique** | Mixed-Effects Model (k = 29; tau^2 estimator: DL)  tau^2 (estimated amount of residual heterogeneity): 0.0416 (SE = 0.0143)  tau (square root of estimated tau^2 value): 0.2039  I^2 (residual heterogeneity / unaccounted variability): 97.86%  H^2 (unaccounted variability / sampling variability): 46.79  R^2 (amount of heterogeneity accounted for): 0.00%  Test for Residual Heterogeneity:  QE(df = 27) = 1263.3119, p-val < .0001  Test of Moderators (coefficient 2):  F(df1 = 1, df2 = 27) = 0.0315, p-val = 0.8606  Model Results:  estimate se tval pval ci.lb ci.ub  intrcpt 0.3834 0.1542 2.4863 0.0194 0.0670 0.6998 *  diagnose -0.0239 0.1347 -0.1773 0.8606 -0.3002 0.2524  ---  Signif. codes: 0 ‘***’ 0.001 ‘**’ 0.01 ‘*’ 0.05 ‘.’ 0.1 ‘ ’ 1 |
| **Continents** | Mixed-Effects Model (k = 29; tau^2 estimator: DL)  tau^2 (estimated amount of residual heterogeneity): 0.0412 (SE = 0.0151)  tau (square root of estimated tau^2 value): 0.2030  I^2 (residual heterogeneity / unaccounted variability): 98.02%  H^2 (unaccounted variability / sampling variability): 50.61  R^2 (amount of heterogeneity accounted for): 0.00%  Test for Residual Heterogeneity:  QE(df = 27) = 1366.4370, p-val < .0001  Test of Moderators (coefficient 2):  F(df1 = 1, df2 = 27) = 0.2703, p-val = 0.6074  Model Results:  estimate se tval pval ci.lb ci.ub  intrcpt 0.3849 0.0674 5.7111 <.0001 0.2466 0.5232 ***  continent -0.0164 0.0316 -0.5199 0.6074 -0.0813 0.0484  ---  Signif. codes: 0 ‘***’ 0.001 ‘**’ 0.01 ‘*’ 0.05 ‘.’ 0.1 ‘ ’ 1 |

**Supplementary table 5. Multi-variable meta-regression for MAFLD prevalence in general population.**

|  | **Results** |
| --- | --- |
| **Multi-variable** | Multimodel Inference: Final Results  --------------------------  - Number of fitted models: 64  - Full formula: ~ deve + income + public + quality + size + continent  - Coefficient significance test: knha  - Interactions modeled: no  - Evaluation criterion: AICc  Best 5 Models  --------------------------  Global model call: metafor::rma(yi = TE, sei = seTE, mods = form, data = glm.data,  method = method, test = test)  ---  Model selection table  (Intrc) cntnn deve publc df logLik AICc delta weight  1 + 2 3.010 -1.6 0.00 0.436  3 + + 3 3.630 -0.3 1.26 0.232  9 + 0.1132 3 3.299 0.4 1.92 0.167  11 + + 0.1165 4 3.996 1.7 3.23 0.087  2 + -0.01653 3 2.544 1.9 3.43 0.078  Models ranked by AICc(x)  Multimodel Inference Coefficients  --------------------------  Estimate Std. Error z value Pr(>\|z\|)  intrcpt 2.698759e-01 0.14671195 1.839495141 0.0658424  deveing 4.420020e-02 0.08044720 0.549431227 0.5827095  public 3.147794e-02 0.06799827 0.462922559 0.6434199  continent -1.948235e-03 0.01362497 0.142990040 0.8862980  quality 3.955227e-03 0.03196101 0.123751635 0.9015119  size 1.113201e-03 0.03474239 0.032041592 0.9744389  incomelower 2.619597e-03 0.03518966 0.074442248 0.9406585  incomeLower 6.260914e-05 0.01585011 0.003950076 0.9968483  incomeUpper 9.113754e-04 0.01297309 0.070251242 0.9439937  Predictor Importance  --------------------------  model importance  1 deve 0.325421133  2 public 0.268868712  3 continent 0.140074951  4 quality 0.132088087  5 size 0.128883790  6 income 0.008530496  > |

**Supplementary Table 6. Leave-one-out analysis for MAFLD prevalence in special population.**

|  | estimate | zval | pval | ci.lb | ci.ub | Q | Qp | tau2 | I2 | H2 |
| --- | --- | --- | --- | --- | --- | --- | --- | --- | --- | --- |
| 1 | 0.4477 | -2.2995 | 0.0215 | 0.404 | 0.4923 | 6007.725 | 0 | 1.0005 | 97.9193 | 48.0618 |
| 2 | 0.4501 | -2.1903 | 0.0285 | 0.4063 | 0.4947 | 5992.482 | 0 | 1.003 | 97.9141 | 47.9399 |
| 3 | 0.4488 | -2.2521 | 0.0243 | 0.4051 | 0.4933 | 6020.855 | 0 | 0.9998 | 97.9239 | 48.1668 |
| 4 | 0.4517 | -2.1348 | 0.0328 | 0.4081 | 0.496 | 5950.592 | 0 | 0.9893 | 97.8994 | 47.6047 |
| 5 | 0.4479 | -2.2823 | 0.0225 | 0.4039 | 0.4926 | 5993.095 | 0 | 1.0103 | 97.9143 | 47.9448 |
| 6 | 0.446 | -2.4102 | 0.0159 | 0.403 | 0.4899 | 5680.418 | 0 | 0.9687 | 97.7995 | 45.4433 |
| 7 | 0.4508 | -2.1689 | 0.0301 | 0.4071 | 0.4952 | 5990.451 | 0 | 0.9957 | 97.9133 | 47.9236 |
| 8 | 0.4485 | -2.2467 | 0.0247 | 0.4043 | 0.4934 | 6015.054 | 0 | 1.0185 | 97.9219 | 48.1204 |
| 9 | 0.4495 | -2.2222 | 0.0263 | 0.4057 | 0.494 | 6017.921 | 0 | 0.9994 | 97.9229 | 48.1434 |
| 10 | 0.4495 | -2.2257 | 0.026 | 0.4058 | 0.4939 | 6019.421 | 0 | 0.9969 | 97.9234 | 48.1554 |
| 11 | 0.4492 | -2.2216 | 0.0263 | 0.4053 | 0.494 | 6017.69 | 0 | 1.0097 | 97.9228 | 48.1415 |
| 12 | 0.4495 | -2.2216 | 0.0263 | 0.4057 | 0.494 | 6017.731 | 0 | 0.9995 | 97.9228 | 48.1418 |
| 13 | 0.4453 | -2.4186 | 0.0156 | 0.4019 | 0.4896 | 5973.246 | 0 | 0.9899 | 97.9073 | 47.786 |
| 14 | 0.445 | -2.4299 | 0.0151 | 0.4016 | 0.4893 | 5987.51 | 0 | 0.9912 | 97.9123 | 47.9001 |
| 15 | 0.4502 | -2.1761 | 0.0295 | 0.4062 | 0.495 | 5942.305 | 0 | 1.0109 | 97.8964 | 47.5384 |
| 16 | 0.4461 | -2.3904 | 0.0168 | 0.4028 | 0.4903 | 5827.139 | 0 | 0.9817 | 97.8549 | 46.6171 |
| 17 | 0.448 | -2.281 | 0.0225 | 0.4042 | 0.4927 | 6011.958 | 0 | 1.0038 | 97.9208 | 48.0957 |
| 18 | 0.4481 | -2.2823 | 0.0225 | 0.4044 | 0.4926 | 6017.544 | 0 | 0.9996 | 97.9227 | 48.1404 |
| 19 | 0.4482 | -2.2799 | 0.0226 | 0.4045 | 0.4927 | 6019.14 | 0 | 0.9983 | 97.9233 | 48.1531 |
| 20 | 0.4475 | -2.3123 | 0.0208 | 0.4038 | 0.492 | 6009.621 | 0 | 0.9979 | 97.92 | 48.077 |
| 21 | 0.447 | -2.3335 | 0.0196 | 0.4034 | 0.4915 | 6000.213 | 0 | 0.9964 | 97.9167 | 48.0017 |
| 22 | 0.4501 | -2.1981 | 0.0279 | 0.4065 | 0.4946 | 6016.913 | 0 | 0.9958 | 97.9225 | 48.1353 |
| 23 | 0.4502 | -2.1911 | 0.0284 | 0.4065 | 0.4947 | 6004.362 | 0 | 0.9986 | 97.9182 | 48.0349 |
| 24 | 0.4493 | -2.223 | 0.0262 | 0.4054 | 0.494 | 6017.512 | 0 | 1.0055 | 97.9227 | 48.1401 |
| 25 | 0.4523 | -2.1245 | 0.0336 | 0.409 | 0.4963 | 5834.193 | 0 | 0.9744 | 97.8575 | 46.6735 |
| 26 | 0.4452 | -2.4233 | 0.0154 | 0.4017 | 0.4895 | 5990.281 | 0 | 0.9916 | 97.9133 | 47.9222 |
| 27 | 0.4484 | -2.2496 | 0.0245 | 0.4042 | 0.4933 | 6010.753 | 0 | 1.0201 | 97.9204 | 48.086 |
| 28 | 0.4489 | -2.2414 | 0.025 | 0.405 | 0.4936 | 6020.804 | 0 | 1.0053 | 97.9239 | 48.1664 |
| 29 | 0.4472 | -2.3272 | 0.02 | 0.4036 | 0.4916 | 6009.013 | 0 | 0.9964 | 97.9198 | 48.0721 |
| 30 | 0.4444 | -2.4615 | 0.0138 | 0.4011 | 0.4886 | 5955.606 | 0 | 0.9866 | 97.9011 | 47.6449 |
| 31 | 0.4494 | -2.2167 | 0.0266 | 0.4055 | 0.4941 | 6015.008 | 0 | 1.0065 | 97.9219 | 48.1201 |
| 32 | 0.451 | -2.8266 | 0.0047 | 0.4175 | 0.4849 | 3046.855 | 0 | 0.5612 | 95.8974 | 24.3748 |
| 33 | 0.4464 | -2.3647 | 0.018 | 0.4029 | 0.4908 | 5966.04 | 0 | 0.9929 | 97.9048 | 47.7283 |
| 34 | 0.4473 | -2.3152 | 0.0206 | 0.4036 | 0.4919 | 5971.094 | 0 | 1.0021 | 97.9066 | 47.7687 |
| 35 | 0.4467 | -2.3516 | 0.0187 | 0.4031 | 0.4911 | 5957.703 | 0 | 0.9944 | 97.9019 | 47.6616 |
| 36 | 0.4461 | -2.3775 | 0.0174 | 0.4026 | 0.4905 | 5989.171 | 0 | 0.993 | 97.9129 | 47.9134 |
| 37 | 0.4489 | -2.2491 | 0.0245 | 0.4051 | 0.4934 | 6020.843 | 0 | 1 | 97.9239 | 48.1667 |
| 38 | 0.4469 | -2.3401 | 0.0193 | 0.4033 | 0.4913 | 5967.471 | 0 | 0.9964 | 97.9053 | 47.7398 |
| 39 | 0.4474 | -2.315 | 0.0206 | 0.4037 | 0.4919 | 5999.617 | 0 | 0.9991 | 97.9165 | 47.9969 |
| 40 | 0.4503 | -2.1884 | 0.0286 | 0.4065 | 0.4948 | 6001.949 | 0 | 0.9985 | 97.9173 | 48.0156 |
| 41 | 0.4493 | -2.2354 | 0.0254 | 0.4056 | 0.4937 | 6020.2 | 0 | 0.9969 | 97.9237 | 48.1616 |
| 42 | 0.4467 | -2.3514 | 0.0187 | 0.4031 | 0.4911 | 6011.145 | 0 | 0.9951 | 97.9205 | 48.0892 |
| 43 | 0.4469 | -2.3424 | 0.0192 | 0.4032 | 0.4913 | 5975.067 | 0 | 0.996 | 97.908 | 47.8005 |
| 44 | 0.4489 | -2.2439 | 0.0248 | 0.4052 | 0.4935 | 6020.723 | 0 | 1.001 | 97.9238 | 48.1658 |
| 45 | 0.4475 | -2.314 | 0.0207 | 0.4038 | 0.4919 | 6012.568 | 0 | 0.9971 | 97.921 | 48.1005 |
| 46 | 0.4484 | -2.2594 | 0.0239 | 0.4044 | 0.4931 | 6015.557 | 0 | 1.0104 | 97.9221 | 48.1245 |
| 47 | 0.4505 | -2.178 | 0.0294 | 0.4068 | 0.495 | 5996.227 | 0 | 0.997 | 97.9154 | 47.9698 |
| 48 | 0.4468 | -2.3468 | 0.0189 | 0.4032 | 0.4912 | 5939.418 | 0 | 0.9952 | 97.8954 | 47.5153 |
| 49 | 0.4481 | -2.2622 | 0.0237 | 0.404 | 0.493 | 6000.681 | 0 | 1.0176 | 97.9169 | 48.0054 |
| 50 | 0.4481 | -2.2841 | 0.0224 | 0.4044 | 0.4926 | 6018.202 | 0 | 0.9986 | 97.923 | 48.1456 |
| 51 | 0.4499 | -2.2052 | 0.0274 | 0.4063 | 0.4944 | 6016.695 | 0 | 0.9965 | 97.9224 | 48.1336 |
| 52 | 0.4469 | -2.342 | 0.0192 | 0.4033 | 0.4913 | 6007.298 | 0 | 0.9955 | 97.9192 | 48.0584 |
| 53 | 0.4492 | -2.2338 | 0.0255 | 0.4055 | 0.4938 | 6019.823 | 0 | 0.9993 | 97.9235 | 48.1586 |
| 54 | 0.4491 | -2.2411 | 0.025 | 0.4055 | 0.4936 | 6020.494 | 0 | 0.997 | 97.9238 | 48.164 |
| 55 | 0.4481 | -2.2827 | 0.0224 | 0.4044 | 0.4926 | 6016.719 | 0 | 1.0002 | 97.9225 | 48.1338 |
| 56 | 0.4497 | -2.2026 | 0.0276 | 0.4058 | 0.4944 | 6004.785 | 0 | 1.008 | 97.9183 | 48.0383 |
| 57 | 0.4481 | -2.2818 | 0.0225 | 0.4043 | 0.4926 | 6014.366 | 0 | 1.0021 | 97.9216 | 48.1149 |
| 58 | 0.4482 | -2.2818 | 0.0225 | 0.4045 | 0.4927 | 6019.279 | 0 | 0.9979 | 97.9233 | 48.1542 |
| 59 | 0.4476 | -2.3076 | 0.021 | 0.4039 | 0.4921 | 6009.936 | 0 | 0.9984 | 97.9201 | 48.0795 |
| 60 | 0.4471 | -2.3315 | 0.0197 | 0.4035 | 0.4915 | 6002.235 | 0 | 0.9965 | 97.9174 | 48.0179 |
| 61 | 0.4475 | -2.3098 | 0.0209 | 0.4039 | 0.492 | 6012.027 | 0 | 0.9977 | 97.9208 | 48.0962 |
| 62 | 0.4498 | -2.212 | 0.027 | 0.4061 | 0.4943 | 6018.025 | 0 | 0.9965 | 97.9229 | 48.1442 |
| 63 | 0.4488 | -2.2531 | 0.0243 | 0.405 | 0.4933 | 6020.84 | 0 | 1.0001 | 97.9239 | 48.1667 |
| 64 | 0.4452 | -2.4256 | 0.0153 | 0.4018 | 0.4894 | 5957.85 | 0 | 0.988 | 97.9019 | 47.6628 |
| 65 | 0.4489 | -2.2409 | 0.025 | 0.4051 | 0.4936 | 6020.737 | 0 | 1.0044 | 97.9238 | 48.1659 |
| 66 | 0.4489 | -2.235 | 0.0254 | 0.405 | 0.4937 | 6020.703 | 0 | 1.0096 | 97.9238 | 48.1656 |
| 67 | 0.4484 | -2.2521 | 0.0243 | 0.4044 | 0.4933 | 6014.481 | 0 | 1.0156 | 97.9217 | 48.1158 |
| 68 | 0.4479 | -2.2914 | 0.0219 | 0.4042 | 0.4924 | 6014.522 | 0 | 0.9996 | 97.9217 | 48.1162 |
| 69 | 0.4469 | -2.3418 | 0.0192 | 0.4033 | 0.4913 | 5998.052 | 0 | 0.9957 | 97.916 | 47.9844 |
| 70 | 0.4497 | -2.2097 | 0.0271 | 0.406 | 0.4943 | 6013.361 | 0 | 1.0003 | 97.9213 | 48.1069 |
| 71 | 0.4486 | -2.2628 | 0.0236 | 0.4049 | 0.4931 | 6020.628 | 0 | 0.9987 | 97.9238 | 48.165 |
| 72 | 0.4488 | -2.2403 | 0.0251 | 0.4048 | 0.4936 | 6020.704 | 0 | 1.0115 | 97.9238 | 48.1656 |
| 73 | 0.4483 | -2.2721 | 0.0231 | 0.4046 | 0.4929 | 6019.212 | 0 | 1.0001 | 97.9233 | 48.1537 |
| 74 | 0.4491 | -2.2366 | 0.0253 | 0.4054 | 0.4937 | 6020.121 | 0 | 1.0002 | 97.9236 | 48.161 |
| 75 | 0.4472 | -2.3248 | 0.0201 | 0.4036 | 0.4917 | 6003.001 | 0 | 0.9972 | 97.9177 | 48.024 |
| 76 | 0.448 | -2.2885 | 0.0221 | 0.4043 | 0.4925 | 6016.424 | 0 | 0.9991 | 97.9224 | 48.1314 |
| 77 | 0.4466 | -2.3574 | 0.0184 | 0.403 | 0.491 | 5992.781 | 0 | 0.9944 | 97.9142 | 47.9422 |
| 78 | 0.4444 | -2.461 | 0.0139 | 0.401 | 0.4886 | 5973.084 | 0 | 0.9888 | 97.9073 | 47.7847 |
| 79 | 0.4496 | -2.2028 | 0.0276 | 0.4056 | 0.4944 | 6005.367 | 0 | 1.0114 | 97.9185 | 48.0429 |
| 80 | 0.4504 | -2.1865 | 0.0288 | 0.4067 | 0.4948 | 6009.915 | 0 | 0.9964 | 97.9201 | 48.0793 |
| 81 | 0.4485 | -2.267 | 0.0234 | 0.4049 | 0.493 | 6020.713 | 0 | 0.9971 | 97.9238 | 48.1657 |
| 82 | 0.448 | -2.2896 | 0.022 | 0.4044 | 0.4925 | 6019.642 | 0 | 0.9966 | 97.9235 | 48.1571 |
| 83 | 0.4476 | -2.3072 | 0.021 | 0.404 | 0.4921 | 6015.512 | 0 | 0.997 | 97.922 | 48.1241 |
| 84 | 0.4483 | -2.2766 | 0.0228 | 0.4047 | 0.4928 | 6020.258 | 0 | 0.9971 | 97.9237 | 48.1621 |
| 85 | 0.4493 | -2.2314 | 0.0257 | 0.4056 | 0.4938 | 6019.858 | 0 | 0.9972 | 97.9235 | 48.1589 |
| 86 | 0.449 | -2.2479 | 0.0246 | 0.4053 | 0.4934 | 6020.73 | 0 | 0.9968 | 97.9238 | 48.1658 |
| 87 | 0.4475 | -2.3113 | 0.0208 | 0.4039 | 0.492 | 6013.144 | 0 | 0.9973 | 97.9212 | 48.1052 |
| 88 | 0.4449 | -2.4332 | 0.015 | 0.4015 | 0.4893 | 5994.052 | 0 | 0.9919 | 97.9146 | 47.9524 |
| 89 | 0.4507 | -2.1721 | 0.0298 | 0.4071 | 0.4952 | 6012.472 | 0 | 0.9951 | 97.921 | 48.0998 |
| 90 | 0.4493 | -2.2308 | 0.0257 | 0.4055 | 0.4938 | 6019.338 | 0 | 1.0005 | 97.9234 | 48.1547 |
| 91 | 0.445 | -2.4371 | 0.0148 | 0.4017 | 0.4892 | 5934.567 | 0 | 0.9849 | 97.8937 | 47.4765 |
| 92 | 0.4463 | -2.3712 | 0.0177 | 0.4027 | 0.4906 | 5999.705 | 0 | 0.9939 | 97.9166 | 47.9976 |
| 93 | 0.4489 | -2.2483 | 0.0246 | 0.4051 | 0.4934 | 6020.835 | 0 | 1.0002 | 97.9239 | 48.1667 |
| 94 | 0.4472 | -2.3259 | 0.02 | 0.4036 | 0.4917 | 6004.682 | 0 | 0.9969 | 97.9183 | 48.0375 |
| 95 | 0.4463 | -2.3707 | 0.0178 | 0.4028 | 0.4907 | 5965.36 | 0 | 0.9922 | 97.9046 | 47.7229 |
| 96 | 0.4484 | -2.2665 | 0.0234 | 0.4046 | 0.493 | 6019.239 | 0 | 1.0019 | 97.9233 | 48.1539 |
| 97 | 0.4494 | -2.2253 | 0.0261 | 0.4057 | 0.494 | 6018.809 | 0 | 0.9984 | 97.9232 | 48.1505 |
| 98 | 0.4498 | -2.2001 | 0.0278 | 0.4059 | 0.4945 | 6002.579 | 0 | 1.0059 | 97.9176 | 48.0206 |
| 99 | 0.4489 | -2.249 | 0.0245 | 0.4052 | 0.4934 | 6020.784 | 0 | 0.9977 | 97.9239 | 48.1663 |
| 100 | 0.447 | -2.3339 | 0.0196 | 0.4034 | 0.4915 | 5999.074 | 0 | 0.9964 | 97.9163 | 47.9926 |
| 101 | 0.4486 | -2.2628 | 0.0236 | 0.4049 | 0.4931 | 6020.628 | 0 | 0.9987 | 97.9238 | 48.165 |
| 102 | 0.4469 | -2.3398 | 0.0193 | 0.4033 | 0.4913 | 5942.727 | 0 | 0.9968 | 97.8966 | 47.5418 |
| 103 | 0.4457 | -2.3995 | 0.0164 | 0.4023 | 0.49 | 5966.272 | 0 | 0.9901 | 97.9049 | 47.7302 |
| 104 | 0.4482 | -2.1915 | 0.0284 | 0.4028 | 0.4945 | 5908.466 | 0 | 1.0843 | 97.8844 | 47.2677 |
| 105 | 0.4469 | -2.3407 | 0.0192 | 0.4033 | 0.4913 | 5972.32 | 0 | 0.9963 | 97.907 | 47.7786 |
| 106 | 0.4494 | -2.2257 | 0.026 | 0.4057 | 0.4939 | 6018.676 | 0 | 0.9991 | 97.9231 | 48.1494 |
| 107 | 0.4482 | -2.2787 | 0.0227 | 0.4045 | 0.4927 | 6018.611 | 0 | 0.9992 | 97.9231 | 48.1489 |
| 108 | 0.4443 | -2.4685 | 0.0136 | 0.401 | 0.4885 | 5940.389 | 0 | 0.9844 | 97.8958 | 47.5231 |
| 109 | 0.4474 | -2.3144 | 0.0206 | 0.4038 | 0.4919 | 6005.644 | 0 | 0.9983 | 97.9186 | 48.0451 |
| 110 | 0.4488 | -2.2513 | 0.0244 | 0.4051 | 0.4934 | 6020.856 | 0 | 0.9997 | 97.9239 | 48.1669 |
| 111 | 0.4467 | -2.3529 | 0.0186 | 0.4031 | 0.4911 | 6010.087 | 0 | 0.995 | 97.9202 | 48.0807 |
| 112 | 0.448 | -2.293 | 0.0218 | 0.4043 | 0.4924 | 6018.744 | 0 | 0.997 | 97.9232 | 48.15 |
| 113 | 0.4474 | -2.3183 | 0.0204 | 0.4037 | 0.4918 | 6010.884 | 0 | 0.997 | 97.9204 | 48.0871 |
| 114 | 0.45 | -2.1975 | 0.028 | 0.4061 | 0.4946 | 6002.095 | 0 | 1.0028 | 97.9174 | 48.0168 |
| 115 | 0.45 | -2.2035 | 0.0276 | 0.4063 | 0.4945 | 6016.586 | 0 | 0.9964 | 97.9224 | 48.1327 |
| 116 | 0.4459 | -2.3901 | 0.0168 | 0.4023 | 0.4902 | 6005.198 | 0 | 0.9938 | 97.9185 | 48.0416 |
| 117 | 0.4457 | -2.3959 | 0.0166 | 0.4022 | 0.4901 | 5995.776 | 0 | 0.9928 | 97.9152 | 47.9662 |
| 118 | 0.4497 | -2.2154 | 0.0267 | 0.4059 | 0.4942 | 6017 | 0 | 0.9982 | 97.9226 | 48.136 |
| 119 | 0.4445 | -2.4543 | 0.0141 | 0.4011 | 0.4888 | 5989.892 | 0 | 0.9911 | 97.9132 | 47.9191 |
| 120 | 0.4462 | -2.3749 | 0.0176 | 0.4027 | 0.4906 | 5973.086 | 0 | 0.9922 | 97.9073 | 47.7847 |
| 121 | 0.446 | -2.383 | 0.0172 | 0.4025 | 0.4904 | 5987.141 | 0 | 0.9926 | 97.9122 | 47.8971 |
| 122 | 0.446 | -2.3825 | 0.0172 | 0.4025 | 0.4904 | 6000.899 | 0 | 0.9936 | 97.917 | 48.0072 |
| 123 | 0.4463 | -2.3721 | 0.0177 | 0.4027 | 0.4906 | 5991.19 | 0 | 0.9934 | 97.9136 | 47.9295 |
| 124 | 0.4459 | -2.3878 | 0.017 | 0.4024 | 0.4903 | 6003.062 | 0 | 0.9936 | 97.9177 | 48.0245 |
| 125 | 0.4525 | -2.0995 | 0.0358 | 0.409 | 0.4968 | 5952.139 | 0 | 0.987 | 97.8999 | 47.6171 |
| 126 | 0.4541 | -2.0801 | 0.0375 | 0.4115 | 0.4973 | 5627.557 | 0 | 0.9379 | 97.7788 | 45.0205 |
| 127 | 0.4477 | -2.3038 | 0.0212 | 0.404 | 0.4922 | 6015.044 | 0 | 0.9975 | 97.9219 | 48.1204 |

**Supplementary Table 7. Leave-one-out diagnostics with a built-in function in MAFLD prevalence in special population.**

|  | **rstudent** | **dffits** | **cook.d** | **cov.r** | **tau2.del** | **QE.del** | **hat** | **weight** | **dfbs** |  |
| --- | --- | --- | --- | --- | --- | --- | --- | --- | --- | --- |
| 1 | 0.195 | 0.017 | 0.0003 | 1.0133 | 1.0005 | 6007.725 | 0.0081 | 0.8075 | 0.017 |  |
| 2 | -0.9817 | -0.0898 | 0.0081 | 1.0157 | 1.003 | 5992.482 | 0.0082 | 0.8152 | -0.0898 |  |
| 3 | -0.346 | -0.0314 | 0.001 | 1.0125 | 0.9998 | 6020.855 | 0.008 | 0.7953 | -0.0314 |  |
| 4 | -1.7931 | -0.1607 | 0.0257 | 1.0024 | 0.9893 | 5950.592 | 0.008 | 0.8026 | -0.1607 |  |
| 5 | 0.1321 | 0.0105 | 0.0001 | 1.0228 | 1.0103 | 5993.095 | 0.0082 | 0.8194 | 0.0105 |  |
| 6 | 0.9875 | 0.0929 | 0.0084 | 0.9826 | 0.9687 | 5680.418 | 0.0082 | 0.8222 | 0.0928 |  |
| 7 | -1.3286 | -0.1194 | 0.0143 | 1.0085 | 0.9957 | 5990.451 | 0.008 | 0.801 | -0.1194 |  |
| 8 | -0.1552 | -0.0164 | 0.0003 | 1.0308 | 1.0185 | 6015.054 | 0.0082 | 0.8207 | -0.0165 |  |
| 9 | -0.6865 | -0.0619 | 0.0038 | 1.012 | 0.9994 | 6017.921 | 0.008 | 0.7955 | -0.0619 |  |
| 10 | -0.7057 | -0.0616 | 0.0038 | 1.0092 | 0.9969 | 6019.421 | 0.0075 | 0.7517 | -0.0616 |  |
| 11 | -0.5471 | -0.0511 | 0.0026 | 1.0222 | 1.0097 | 6017.69 | 0.0082 | 0.817 | -0.0511 |  |
| 12 | -0.6925 | -0.0625 | 0.0039 | 1.0121 | 0.9995 | 6017.731 | 0.008 | 0.7963 | -0.0625 |  |
| 13 | 1.4039 | 0.1245 | 0.0154 | 1.0027 | 0.9899 | 5973.246 | 0.0077 | 0.7738 | 0.1245 |  |
| 14 | 1.585 | 0.1368 | 0.0187 | 1.0035 | 0.9912 | 5987.51 | 0.0074 | 0.7356 | 0.1368 |  |
| 15 | -1.0314 | -0.0954 | 0.0092 | 1.0234 | 1.0109 | 5942.305 | 0.0082 | 0.8217 | -0.0954 |  |
| 16 | 0.9482 | 0.0877 | 0.0076 | 0.9952 | 0.9817 | 5827.139 | 0.0082 | 0.8194 | 0.0876 |  |
| 17 | 0.0337 | 0.0022 | 0 | 1.0165 | 1.0038 | 6011.958 | 0.0081 | 0.8124 | 0.0022 |  |
| 18 | -0.0105 | -0.0014 | 0 | 1.0122 | 0.9996 | 6017.544 | 0.008 | 0.7972 | -0.0014 |  |
| 19 | -0.0551 | -0.0052 | 0 | 1.0109 | 0.9983 | 6019.14 | 0.0078 | 0.7844 | -0.0052 |  |
| 20 | 0.3031 | 0.0269 | 0.0007 | 1.0106 | 0.9979 | 6009.621 | 0.0079 | 0.795 | 0.0269 |  |
| 21 | 0.522 | 0.0466 | 0.0022 | 1.0092 | 0.9964 | 6000.213 | 0.008 | 0.796 | 0.0466 |  |
| 22 | -1.0712 | -0.0909 | 0.0083 | 1.0078 | 0.9958 | 6016.913 | 0.0071 | 0.7139 | -0.0909 |  |
| 23 | -1.0408 | -0.094 | 0.0089 | 1.0113 | 0.9986 | 6004.362 | 0.008 | 0.8031 | -0.094 |  |
| 24 | -0.5874 | -0.0542 | 0.003 | 1.0182 | 1.0055 | 6017.512 | 0.0081 | 0.8134 | -0.0542 |  |
| 25 | -2.095 | -0.1877 | 0.0345 | 0.9881 | 0.9744 | 5834.193 | 0.0081 | 0.8136 | -0.1876 |  |
| 26 | 1.5147 | 0.1306 | 0.017 | 1.0039 | 0.9916 | 5990.281 | 0.0073 | 0.7344 | 0.1306 |  |
| 27 | -0.1031 | -0.0119 | 0.0001 | 1.0323 | 1.0201 | 6010.753 | 0.0082 | 0.8212 | -0.0119 |  |
| 28 | -0.3867 | -0.036 | 0.0013 | 1.0179 | 1.0053 | 6020.804 | 0.0081 | 0.8123 | -0.036 |  |
| 29 | 0.4531 | 0.0401 | 0.0016 | 1.009 | 0.9964 | 6009.013 | 0.0078 | 0.7817 | 0.0401 |  |
| 30 | 1.8548 | 0.1638 | 0.0266 | 0.9994 | 0.9866 | 5955.606 | 0.0077 | 0.766 | 0.1638 |  |
| 31 | -0.6436 | -0.0595 | 0.0036 | 1.0191 | 1.0065 | 6015.008 | 0.0081 | 0.8149 | -0.0595 |  |
| 32 | -2.8726 | -0.1715 | 0.0166 | 0.5887 | 0.5612 | 3046.855 | 0.0083 | 0.8256 | -0.1686 |  |
| 33 | 0.8194 | 0.0741 | 0.0055 | 1.0058 | 0.9929 | 5966.04 | 0.0081 | 0.8059 | 0.0741 |  |
| 34 | 0.3896 | 0.0347 | 0.0012 | 1.0149 | 1.0021 | 5971.094 | 0.0082 | 0.8173 | 0.0347 |  |
| 35 | 0.6934 | 0.0628 | 0.0039 | 1.0074 | 0.9944 | 5957.703 | 0.0081 | 0.8122 | 0.0628 |  |
| 36 | 0.9761 | 0.0868 | 0.0075 | 1.0057 | 0.993 | 5989.171 | 0.0078 | 0.781 | 0.0868 |  |
| 37 | -0.3766 | -0.0342 | 0.0012 | 1.0126 | 1 | 6020.843 | 0.008 | 0.7963 | -0.0342 |  |
| 38 | 0.5916 | 0.0534 | 0.0029 | 1.0094 | 0.9964 | 5967.471 | 0.0081 | 0.8127 | 0.0534 |  |
| 39 | 0.3489 | 0.0311 | 0.001 | 1.0119 | 0.9991 | 5999.617 | 0.0081 | 0.8073 | 0.0311 |  |
| 40 | -1.0714 | -0.0968 | 0.0094 | 1.0112 | 0.9985 | 6001.949 | 0.008 | 0.8043 | -0.0968 |  |
| 41 | -0.599 | -0.052 | 0.0027 | 1.0091 | 0.9969 | 6020.2 | 0.0074 | 0.7442 | -0.052 |  |
| 42 | 0.7264 | 0.0623 | 0.0039 | 1.0072 | 0.9951 | 6011.145 | 0.0073 | 0.7305 | 0.0623 |  |
| 43 | 0.6112 | 0.0551 | 0.003 | 1.0089 | 0.996 | 5975.067 | 0.0081 | 0.8097 | 0.0551 |  |
| 44 | -0.4181 | -0.0382 | 0.0015 | 1.0137 | 1.001 | 6020.723 | 0.008 | 0.802 | -0.0382 |  |
| 45 | 0.3127 | 0.0276 | 0.0008 | 1.0097 | 0.9971 | 6012.568 | 0.0078 | 0.7828 | 0.0276 |  |
| 46 | -0.1192 | -0.0124 | 0.0002 | 1.0229 | 1.0104 | 6015.557 | 0.0082 | 0.8177 | -0.0124 |  |
| 47 | -1.2083 | -0.1088 | 0.0119 | 1.0098 | 0.997 | 5996.227 | 0.008 | 0.8023 | -0.1088 |  |
| 48 | 0.6492 | 0.0589 | 0.0035 | 1.0083 | 0.9952 | 5939.418 | 0.0082 | 0.8163 | 0.0589 |  |
| 49 | 0.0061 | -0.0017 | 0 | 1.0299 | 1.0176 | 6000.681 | 0.0082 | 0.8211 | -0.0017 |  |
| 50 | -0.0041 | -0.0007 | 0 | 1.0112 | 0.9986 | 6018.202 | 0.0079 | 0.7889 | -0.0007 |  |
| 51 | -0.9435 | -0.0825 | 0.0068 | 1.0089 | 0.9965 | 6016.695 | 0.0076 | 0.7559 | -0.0825 |  |
| 52 | 0.6104 | 0.0538 | 0.0029 | 1.0081 | 0.9955 | 6007.298 | 0.0077 | 0.7709 | 0.0538 |  |
| 53 | -0.5599 | -0.0505 | 0.0026 | 1.0119 | 0.9993 | 6019.823 | 0.0079 | 0.7925 | -0.0505 |  |
| 54 | -0.5296 | -0.0462 | 0.0021 | 1.0092 | 0.997 | 6020.494 | 0.0075 | 0.7479 | -0.0462 |  |
| 55 | 0.0029 | -0.0003 | 0 | 1.0129 | 1.0002 | 6016.719 | 0.008 | 0.801 | -0.0003 |  |
| 56 | -0.7785 | -0.0719 | 0.0052 | 1.0206 | 1.008 | 6004.785 | 0.0082 | 0.8174 | -0.072 |  |
| 57 | 0.0198 | 0.0011 | 0 | 1.0148 | 1.0021 | 6014.366 | 0.0081 | 0.8085 | 0.0011 |  |
| 58 | -0.0418 | -0.004 | 0 | 1.0104 | 0.9979 | 6019.279 | 0.0078 | 0.7771 | -0.004 |  |
| 59 | 0.2582 | 0.0228 | 0.0005 | 1.0112 | 0.9984 | 6009.936 | 0.008 | 0.7983 | 0.0228 |  |
| 60 | 0.5005 | 0.0446 | 0.002 | 1.0093 | 0.9965 | 6002.235 | 0.0079 | 0.7943 | 0.0446 |  |
| 61 | 0.2726 | 0.0241 | 0.0006 | 1.0103 | 0.9977 | 6012.027 | 0.0079 | 0.7901 | 0.0241 |  |
| 62 | -0.8755 | -0.0759 | 0.0058 | 1.0087 | 0.9965 | 6018.025 | 0.0074 | 0.7441 | -0.0759 |  |
| 63 | -0.3298 | -0.0301 | 0.0009 | 1.0128 | 1.0001 | 6020.84 | 0.008 | 0.7972 | -0.0301 |  |
| 64 | 1.4492 | 0.1296 | 0.0167 | 1.0009 | 0.988 | 5957.85 | 0.0078 | 0.7845 | 0.1296 |  |
| 65 | -0.4038 | -0.0374 | 0.0014 | 1.0171 | 1.0044 | 6020.737 | 0.0081 | 0.811 | -0.0374 |  |
| 66 | -0.3995 | -0.0377 | 0.0014 | 1.0221 | 1.0096 | 6020.703 | 0.0082 | 0.8166 | -0.0377 |  |
| 67 | -0.132 | -0.014 | 0.0002 | 1.028 | 1.0156 | 6014.481 | 0.0082 | 0.8199 | -0.0141 |  |
| 68 | 0.0933 | 0.0079 | 0.0001 | 1.0124 | 0.9996 | 6014.522 | 0.008 | 0.8004 | 0.0079 |  |
| 69 | 0.6051 | 0.0541 | 0.0029 | 1.0085 | 0.9957 | 5998.052 | 0.0079 | 0.7934 | 0.0541 |  |
| 70 | -0.8101 | -0.0734 | 0.0054 | 1.013 | 1.0003 | 6013.361 | 0.008 | 0.8038 | -0.0734 |  |
| 71 | -0.2443 | -0.0221 | 0.0005 | 1.0112 | 0.9987 | 6020.628 | 0.0079 | 0.7851 | -0.0221 |  |
| 72 | -0.3166 | -0.0304 | 0.0009 | 1.024 | 1.0115 | 6020.704 | 0.0082 | 0.8178 | -0.0304 |  |
| 73 | -0.1178 | -0.0111 | 0.0001 | 1.0127 | 1.0001 | 6019.212 | 0.008 | 0.7985 | -0.0111 |  |
| 74 | -0.5129 | -0.0465 | 0.0022 | 1.0129 | 1.0002 | 6020.121 | 0.008 | 0.7983 | -0.0465 |  |
| 75 | 0.4341 | 0.0387 | 0.0015 | 1.01 | 0.9972 | 6003.001 | 0.008 | 0.7979 | 0.0387 |  |
| 76 | 0.0534 | 0.0044 | 0 | 1.0118 | 0.9991 | 6016.424 | 0.008 | 0.7957 | 0.0044 |  |
| 77 | 0.7642 | 0.0683 | 0.0047 | 1.0072 | 0.9944 | 5992.781 | 0.0079 | 0.7901 | 0.0683 |  |
| 78 | 1.9095 | 0.1655 | 0.0272 | 1.0013 | 0.9888 | 5973.084 | 0.0074 | 0.7399 | 0.1655 |  |
| 79 | -0.7313 | -0.0681 | 0.0047 | 1.0239 | 1.0114 | 6005.367 | 0.0082 | 0.819 | -0.0681 |  |
| 80 | -1.139 | -0.1011 | 0.0102 | 1.0091 | 0.9964 | 6009.915 | 0.0078 | 0.7799 | -0.1011 |  |
| 81 | -0.2277 | -0.02 | 0.0004 | 1.0093 | 0.9971 | 6020.713 | 0.0075 | 0.7495 | -0.02 |  |
| 82 | 0.0247 | 0.002 | 0 | 1.0088 | 0.9966 | 6019.642 | 0.0074 | 0.7362 | 0.002 |  |
| 83 | 0.2357 | 0.0206 | 0.0004 | 1.0096 | 0.997 | 6015.512 | 0.0077 | 0.7739 | 0.0206 |  |
| 84 | -0.1154 | -0.0103 | 0.0001 | 1.0094 | 0.9971 | 6020.258 | 0.0075 | 0.7542 | -0.0103 |  |
| 85 | -0.6325 | -0.0555 | 0.0031 | 1.0095 | 0.9972 | 6019.858 | 0.0076 | 0.7589 | -0.0555 |  |
| 86 | -0.4594 | -0.0397 | 0.0016 | 1.0089 | 0.9968 | 6020.73 | 0.0073 | 0.7344 | -0.0397 |  |
| 87 | 0.2844 | 0.0251 | 0.0006 | 1.0099 | 0.9973 | 6013.144 | 0.0078 | 0.7835 | 0.0251 |  |
| 88 | 1.6639 | 0.1405 | 0.0197 | 1.0039 | 0.9919 | 5994.052 | 0.007 | 0.7047 | 0.1405 |  |
| 89 | -1.3829 | -0.1177 | 0.0139 | 1.0072 | 0.9951 | 6012.472 | 0.0072 | 0.7195 | -0.1177 |  |
| 90 | -0.5731 | -0.052 | 0.0027 | 1.0132 | 1.0005 | 6019.338 | 0.008 | 0.8007 | -0.052 |  |
| 91 | 1.5292 | 0.1378 | 0.0188 | 0.998 | 0.9849 | 5934.567 | 0.0079 | 0.7931 | 0.1378 |  |
| 92 | 0.9249 | 0.0813 | 0.0066 | 1.0065 | 0.9939 | 5999.705 | 0.0076 | 0.7638 | 0.0813 |  |
| 93 | -0.3818 | -0.0348 | 0.0012 | 1.0129 | 1.0002 | 6020.835 | 0.008 | 0.7977 | -0.0348 |  |
| 94 | 0.4431 | 0.0395 | 0.0016 | 1.0097 | 0.9969 | 6004.682 | 0.0079 | 0.7943 | 0.0395 |  |
| 95 | 0.8777 | 0.0793 | 0.0063 | 1.0052 | 0.9922 | 5965.36 | 0.008 | 0.8041 | 0.0793 |  |
| 96 | -0.1544 | -0.0146 | 0.0002 | 1.0146 | 1.0019 | 6019.239 | 0.0081 | 0.8059 | -0.0146 |  |
| 97 | -0.6714 | -0.0601 | 0.0036 | 1.011 | 0.9984 | 6018.809 | 0.0079 | 0.7855 | -0.0601 |  |
| 98 | -0.8344 | -0.0768 | 0.006 | 1.0186 | 1.0059 | 6002.579 | 0.0082 | 0.8162 | -0.0768 |  |
| 99 | -0.4199 | -0.0372 | 0.0014 | 1.0101 | 0.9977 | 6020.784 | 0.0077 | 0.7678 | -0.0372 |  |
| 100 | 0.5261 | 0.047 | 0.0022 | 1.0092 | 0.9964 | 5999.074 | 0.008 | 0.7973 | 0.047 |  |
| 101 | -0.2443 | -0.0221 | 0.0005 | 1.0112 | 0.9987 | 6020.628 | 0.0079 | 0.7851 | -0.0221 |  |
| 102 | 0.5925 | 0.0536 | 0.0029 | 1.0098 | 0.9968 | 5942.727 | 0.0082 | 0.817 | 0.0536 |  |
| 103 | 1.178 | 0.1058 | 0.0111 | 1.003 | 0.9901 | 5966.272 | 0.0079 | 0.7914 | 0.1058 |  |
| 104 | 0.0366 | -0.0048 | 0 | 1.0943 | 1.0843 | 5908.466 | 0.0083 | 0.8254 | -0.0048 |  |
| 105 | 0.5965 | 0.0538 | 0.0029 | 1.0092 | 0.9963 | 5972.32 | 0.0081 | 0.8111 | 0.0538 |  |
| 106 | -0.6536 | -0.0588 | 0.0035 | 1.0117 | 0.9991 | 6018.676 | 0.0079 | 0.7925 | -0.0588 |  |
| 107 | -0.0549 | -0.0053 | 0 | 1.0119 | 0.9992 | 6018.611 | 0.0079 | 0.7939 | -0.0053 |  |
| 108 | 1.8941 | 0.1686 | 0.0281 | 0.9974 | 0.9844 | 5940.389 | 0.0078 | 0.7756 | 0.1686 |  |
| 109 | 0.3319 | 0.0295 | 0.0009 | 1.011 | 0.9983 | 6005.644 | 0.008 | 0.801 | 0.0295 |  |
| 110 | -0.3566 | -0.0324 | 0.0011 | 1.0124 | 0.9997 | 6020.856 | 0.0079 | 0.7946 | -0.0324 |  |
| 111 | 0.7403 | 0.0638 | 0.0041 | 1.0072 | 0.995 | 6010.087 | 0.0074 | 0.7374 | 0.0638 |  |
| 112 | 0.0721 | 0.0061 | 0 | 1.0093 | 0.997 | 6018.744 | 0.0076 | 0.7586 | 0.0061 |  |
| 113 | 0.3592 | 0.0318 | 0.001 | 1.0096 | 0.997 | 6010.884 | 0.0078 | 0.7849 | 0.0318 |  |
| 114 | -0.9057 | -0.0828 | 0.0069 | 1.0156 | 1.0028 | 6002.095 | 0.0081 | 0.8133 | -0.0828 |  |
| 115 | -0.9692 | -0.0845 | 0.0071 | 1.0087 | 0.9964 | 6016.586 | 0.0075 | 0.7514 | -0.0845 |  |
| 116 | 1.1771 | 0.0995 | 0.0099 | 1.0057 | 0.9938 | 6005.198 | 0.0071 | 0.707 | 0.0995 |  |
| 117 | 1.2023 | 0.1046 | 0.0109 | 1.0052 | 0.9928 | 5995.776 | 0.0075 | 0.7485 | 0.1046 |  |
| 118 | -0.7841 | -0.0701 | 0.0049 | 1.0108 | 0.9982 | 6017 | 0.0079 | 0.7867 | -0.0701 |  |
| 119 | 1.9189 | 0.1606 | 0.0257 | 1.003 | 0.9911 | 5989.892 | 0.0069 | 0.6928 | 0.1607 |  |
| 120 | 0.9277 | 0.0835 | 0.007 | 1.0052 | 0.9922 | 5973.086 | 0.008 | 0.7983 | 0.0835 |  |
| 121 | 1.0333 | 0.0919 | 0.0084 | 1.0053 | 0.9926 | 5987.141 | 0.0078 | 0.78 | 0.0919 |  |
| 122 | 1.0607 | 0.0921 | 0.0085 | 1.006 | 0.9936 | 6000.899 | 0.0075 | 0.7456 | 0.0921 |  |
| 123 | 0.9201 | 0.0819 | 0.0067 | 1.0061 | 0.9934 | 5991.19 | 0.0078 | 0.7819 | 0.0819 |  |
| 124 | 1.1342 | 0.0972 | 0.0094 | 1.0058 | 0.9936 | 6003.062 | 0.0073 | 0.7266 | 0.0972 |  |
| 125 | -2.248 | -0.199 | 0.0393 | 0.9999 | 0.987 | 5952.139 | 0.0078 | 0.784 | -0.199 |  |
| 126 | -3.1058 | -0.275 | 0.0714 | 0.9528 | 0.9379 | 5627.557 | 0.0081 | 0.8118 | -0.2748 | * |
| 127 | 0.2039 | 0.0179 | 0.0003 | 1.0101 | 0.9975 | 6015.044 | 0.0078 | 0.783 | 0.0179 |  |

**Supplementary Table 8. Univariate meta-regression for MAFLD prevalence in special population**

| **Covariate** | **Results** |
| --- | --- |
| **Size** | Mixed-Effects Model (k = 127; tau^2 estimator: DL)  tau^2 (estimated amount of residual heterogeneity): 0.0455 (SE = 0.0244)  tau (square root of estimated tau^2 value): 0.2134  I^2 (residual heterogeneity / unaccounted variability): 98.80%  H^2 (unaccounted variability / sampling variability): 83.37  R^2 (amount of heterogeneity accounted for): 12.89%  Test for Residual Heterogeneity:  QE(df = 125) = 10421.4180, p-val < .0001  Test of Moderators (coefficient 2):  F(df1 = 1, df2 = 125) = 9.4983, p-val = 0.0025  Model Results:  estimate se tval pval ci.lb ci.ub  intrcpt 0.6308 0.0588 10.7338 <.0001 0.5145 0.7471 ***  size -0.1063 0.0345 -3.0819 0.0025 -0.1746 -0.0380 **  ---  Signif. codes: 0 ‘***’ 0.001 ‘**’ 0.01 ‘*’ 0.05 ‘.’ 0.1 ‘ ’ 1 |
| **Continents** | Mixed-Effects Model (k = 127; tau^2 estimator: DL)  tau^2 (estimated amount of residual heterogeneity): 0.0572 (SE = 0.0205)  tau (square root of estimated tau^2 value): 0.2392  I^2 (residual heterogeneity / unaccounted variability): 98.68%  H^2 (unaccounted variability / sampling variability): 75.75  R^2 (amount of heterogeneity accounted for): 0.00%  Test for Residual Heterogeneity:  QE(df = 121) = 9165.8563, p-val < .0001  Test of Moderators (coefficients 2:6):  F(df1 = 5, df2 = 121) = 2.2829, p-val = 0.0506  Model Results:  estimate se tval pval ci.lb ci.ub  intrcpt 0.5045 0.0258 19.5528 <.0001 0.4534 0.5555 ***  contiEurope -0.1135 0.0394 -2.8780 0.0047 -0.1915 -0.0354 **  contiAfrica 0.0156 0.0993 0.1570 0.8755 -0.1809 0.2121  contiNorth America -0.0250 0.0526 -0.4753 0.6354 -0.1292 0.0792  contiOceania 0.1700 0.1896 0.8965 0.3718 -0.2054 0.5453  contiSouth America -0.1054 0.0606 -1.7393 0.0845 -0.2254 0.0146 .  ---  Signif. codes: 0 ‘***’ 0.001 ‘**’ 0.01 ‘*’ 0.05 ‘.’ 0.1 ‘ ’ 1 |
| **Country development** | Mixed-Effects Model (k = 127; tau^2 estimator: DL)  tau^2 (estimated amount of residual heterogeneity): 0.0444 (SE = 0.0230)  tau (square root of estimated tau^2 value): 0.2107  I^2 (residual heterogeneity / unaccounted variability): 98.68%  H^2 (unaccounted variability / sampling variability): 75.90  R^2 (amount of heterogeneity accounted for): 15.04%  Test for Residual Heterogeneity:  QE(df = 125) = 9487.8082, p-val < .0001  Test of Moderators (coefficient 2):  F(df1 = 1, df2 = 125) = 3.2654, p-val = 0.0732  Model Results:  estimate se tval pval ci.lb ci.ub  intrcpt 0.4256 0.0243 17.4860 <.0001 0.3774 0.4738 ***  deveing 0.0615 0.0340 1.8071 0.0732 -0.0059 0.1288 .  ---  Signif. codes: 0 ‘***’ 0.001 ‘**’ 0.01 ‘*’ 0.05 ‘.’ 0.1 ‘ ’ 1 |
| **Country or regional income** | Mixed-Effects Model (k = 127; tau^2 estimator: DL)  tau^2 (estimated amount of residual heterogeneity): 0.0470 (SE = 0.0249)  tau (square root of estimated tau^2 value): 0.2168  I^2 (residual heterogeneity / unaccounted variability): 98.75%  H^2 (unaccounted variability / sampling variability): 79.77  R^2 (amount of heterogeneity accounted for): 10.03%  Test for Residual Heterogeneity:  QE(df = 124) = 9890.9609, p-val < .0001  Test of Moderators (coefficients 2:3):  F(df1 = 2, df2 = 124) = 0.7224, p-val = 0.4876  Model Results:  estimate se tval pval ci.lb ci.ub  intrcpt 0.4376 0.0237 18.4914 <.0001 0.3908 0.4844 ***  incomelower 0.0435 0.0832 0.5229 0.6020 -0.1211 0.2081  incomeupper 0.0412 0.0354 1.1620 0.2475 -0.0290 0.1113  ---  Signif. codes: 0 ‘***’ 0.001 ‘**’ 0.01 ‘*’ 0.05 ‘.’ 0.1 ‘ ’ 1 |
| **Publication time** | Mixed-Effects Model (k = 127; tau^2 estimator: DL)  tau^2 (estimated amount of residual heterogeneity): 0.0576 (SE = 0.0196)  tau (square root of estimated tau^2 value): 0.2399  I^2 (residual heterogeneity / unaccounted variability): 98.72%  H^2 (unaccounted variability / sampling variability): 78.26  R^2 (amount of heterogeneity accounted for): 0.00%  Test for Residual Heterogeneity:  QE(df = 125) = 9782.7312, p-val < .0001  Test of Moderators (coefficient 2):  F(df1 = 1, df2 = 125) = 0.0171, p-val = 0.8963  Model Results:  estimate se tval pval ci.lb ci.ub  intrcpt 0.4671 0.0762 6.1264 <.0001 0.3162 0.6180 ***  public -0.0054 0.0417 -0.1306 0.8963 -0.0879 0.0771  ---  Signif. codes: 0 ‘***’ 0.001 ‘**’ 0.01 ‘*’ 0.05 ‘.’ 0.1 ‘ ’ 1 |
| **Study quality** | Mixed-Effects Model (k = 127; tau^2 estimator: DL)  tau^2 (estimated amount of residual heterogeneity): 0.0617 (SE = 0.0260)  tau (square root of estimated tau^2 value): 0.2483  I^2 (residual heterogeneity / unaccounted variability): 98.94%  H^2 (unaccounted variability / sampling variability): 94.28  R^2 (amount of heterogeneity accounted for): 0.00%  Test for Residual Heterogeneity:  QE(df = 125) = 11785.3485, p-val < .0001  Test of Moderators (coefficient 2):  F(df1 = 1, df2 = 125) = 0.7492, p-val = 0.3884  Model Results:  estimate se tval pval ci.lb ci.ub  intrcpt 0.5008 0.0529 9.4589 <.0001 0.3960 0.6056 ***  quality -0.0299 0.0345 -0.8655 0.3884 -0.0981 0.0384  ---  Signif. codes: 0 ‘***’ 0.001 ‘**’ 0.01 ‘*’ 0.05 ‘.’ 0.1 ‘ ’ 1 |
| **Diagnostic techniques** | Mixed-Effects Model (k = 127; tau^2 estimator: DL)  tau^2 (estimated amount of residual heterogeneity): 0.0607 (SE = 0.0289)  tau (square root of estimated tau^2 value): 0.2464  I^2 (residual heterogeneity / unaccounted variability): 98.95%  H^2 (unaccounted variability / sampling variability): 95.20  R^2 (amount of heterogeneity accounted for): 0.00%  Test for Residual Heterogeneity:  QE(df = 125) = 11899.7124, p-val < .0001  Test of Moderators (coefficient 2):  F(df1 = 1, df2 = 125) = 0.0801, p-val = 0.7777  Model Results:  estimate se tval pval ci.lb ci.ub  intrcpt 0.4645 0.0301 15.4259 <.0001 0.4049 0.5241 ***  diagnose -0.0047 0.0168 -0.2830 0.7777 -0.0379 0.0284  ---  Signif. codes: 0 ‘***’ 0.001 ‘**’ 0.01 ‘*’ 0.05 ‘.’ 0.1 ‘ ’ 1 |

**Supplementary Table 9. Multi-variable meta-regression for MAFLD prevalence in special population**

|  | **Results** |
| --- | --- |
| **Multi-variable** | **Multimodel Inference: Final Results**  **--------------------------**  **- Number of fitted models: 128**  **- Full formula: ~ deve + income + public + quality + size + conti + diagnose**  **- Coefficient significance test: knha**  **- Interactions modeled: no**  **- Evaluation criterion: AICc**  **Best 5 Models**  **--------------------------**  **Global model call: metafor::rma(yi = TE, sei = seTE, mods = form, data = glm.data,**  **method = method, test = test)**  **---**  **Model selection table**  **(Intrc) conti deve qulty size df logLik AICc delta weight**  **65 + + 3 32.581 -59.0 0.00 0.276**  **67 + + + 4 33.623 -58.9 0.05 0.269**  **66 + -0.02724 + 4 33.459 -58.6 0.38 0.228**  **68 + -0.02387 + + 5 34.115 -57.7 1.23 0.149**  **99 + + 0.0281 + 5 33.464 -56.4 2.53 0.078**  **Models ranked by AICc(x)**  **Multimodel Inference Coefficients**  **--------------------------**  **Estimate Std. Error z value Pr(>\|z\|)**  **intrcpt 0.4888882167 0.130457304 3.74749594 0.0001786**  **size2 -0.1176816661 0.041084704 2.86436690 0.0041784**  **deveing 0.0254540480 0.037258901 0.68316690 0.4945014**  **conti -0.0106789846 0.016159772 0.66083760 0.5087165**  **quality 0.0055699199 0.016202226 0.34377498 0.7310155**  **public 0.0051650988 0.021225010 0.24334965 0.8077345**  **diagnose -0.0004576959 0.004704151 0.09729619 0.9224912**  **incomelower 0.0063880199 0.032298585 0.19778018 0.8432171**  **incomeupper 0.0042703786 0.018819250 0.22691545 0.8204895**  **Predictor Importance**  **--------------------------**  **model importance**  **1 size 0.97364676**  **2 deve 0.42630995**  **3 conti 0.40732346**  **4 quality 0.21071825**  **5 public 0.17187863**  **6 diagnose 0.14549356**  **7 income 0.07978829**  **>** |

**Supplementary Table 10. Egger’s test for included studies.**

| Groups | P value |
| --- | --- |
| Studies in general population regardless of diagnostic technique | <0.01 |
| Studies in general population diagnosed by ultrasound | 0.39 |
| Studies in special population from clinics regardless of diagnostic technique | <0.01 |
| Studies in special population from clinics diagnosed by ultrasound | <0.01 |

**Supplementary Figure 1. Leave-one-out analysis for MAFLD prevalence in general population.**

**
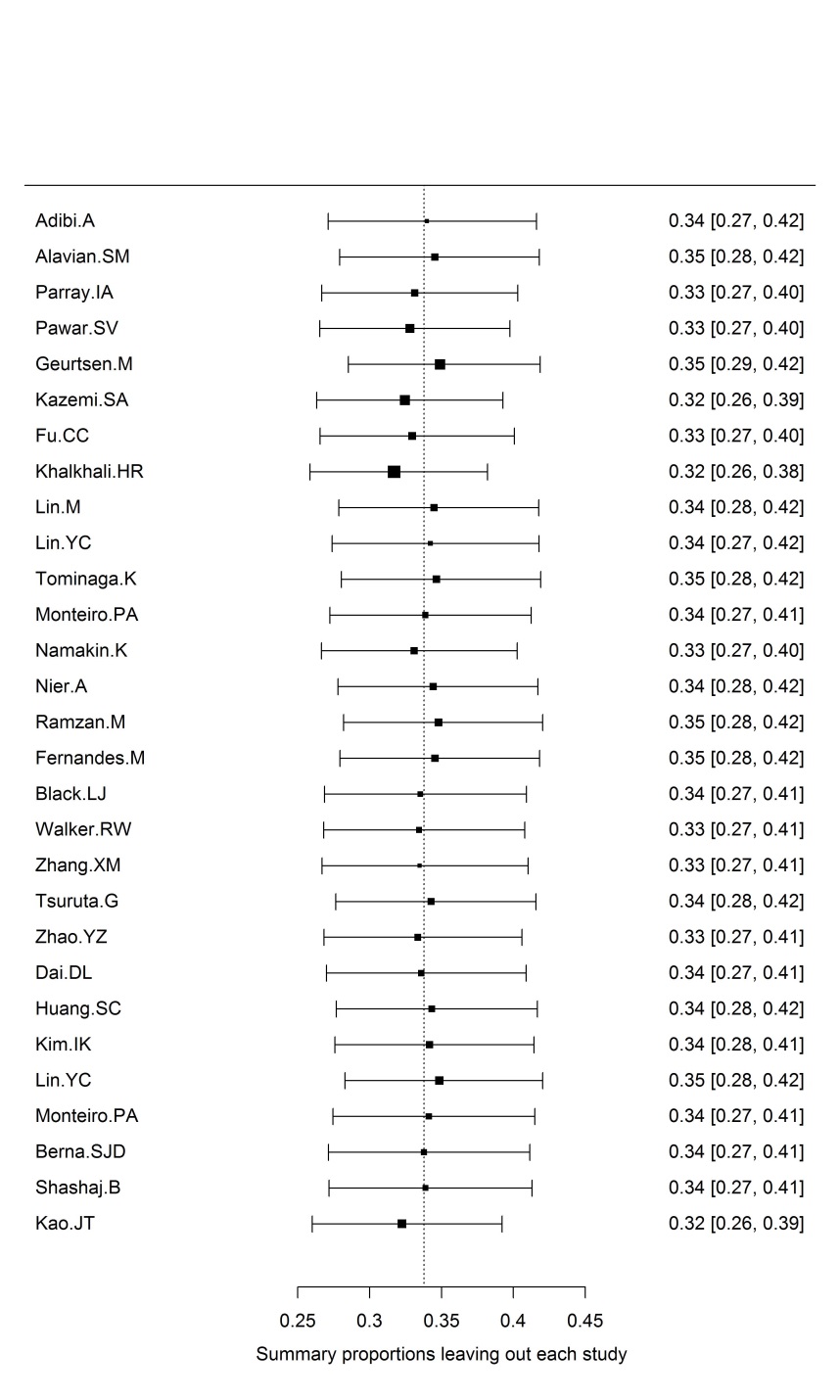
**

**Supplementary figure 2. Leave-one-out diagnostics with a built-in function in MAFLD prevalence in general population.**

**
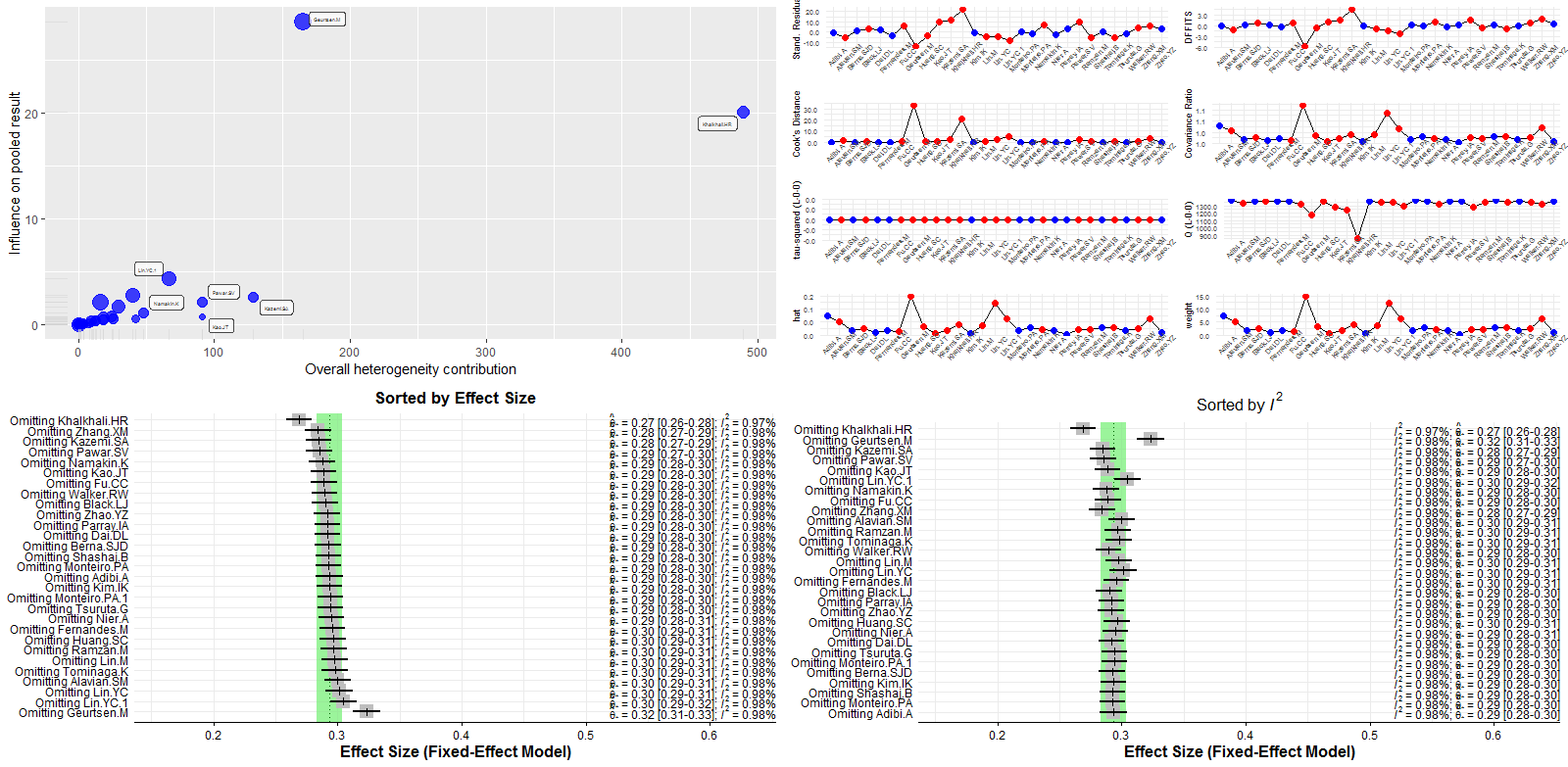
**

**Supplementary Figure 3. Leave-one-out analysis for MAFLD prevalence in special population.**

**
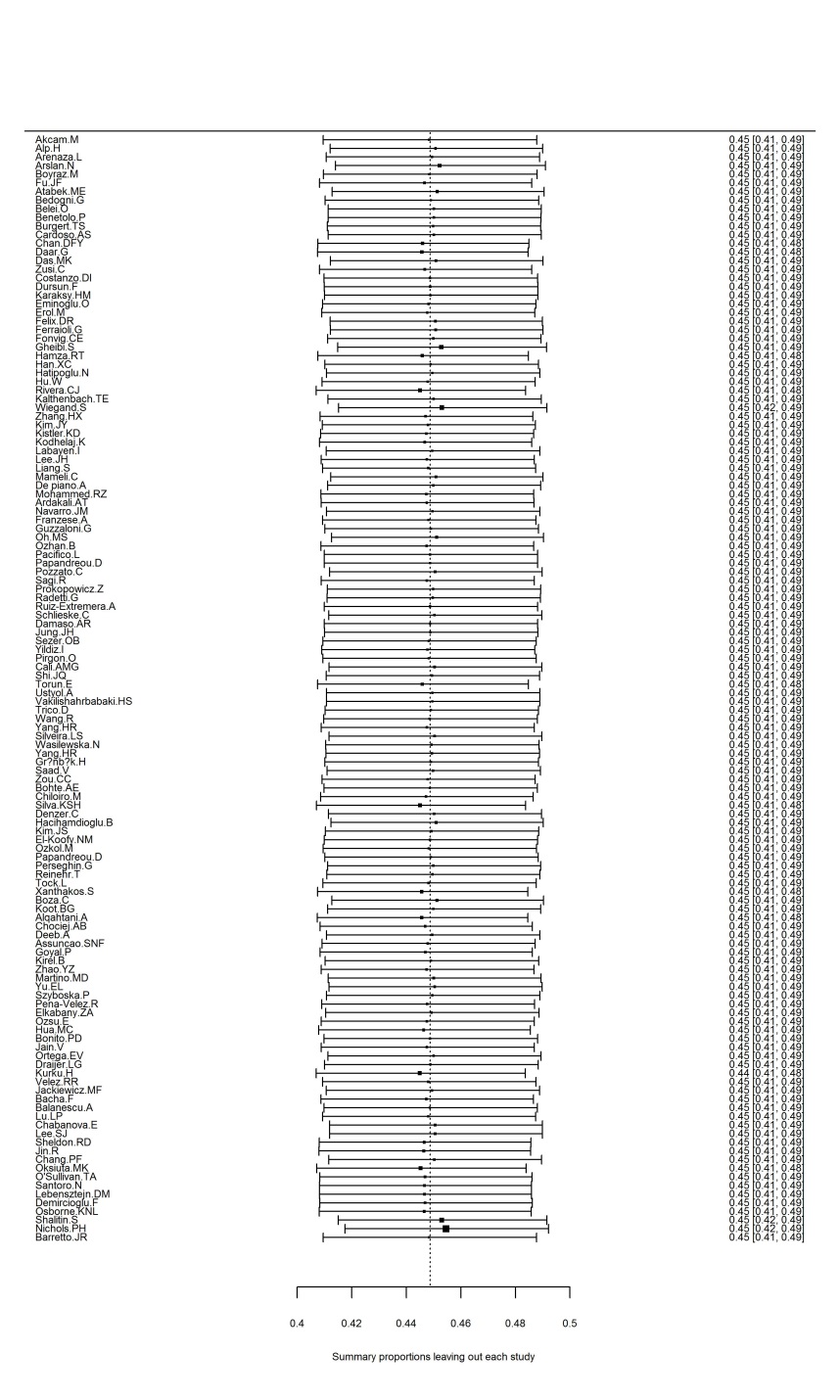
**

**Supplementary figure 4. Leave-one-out diagnostics with a built-in function in MAFLD prevalence in special population.**

**
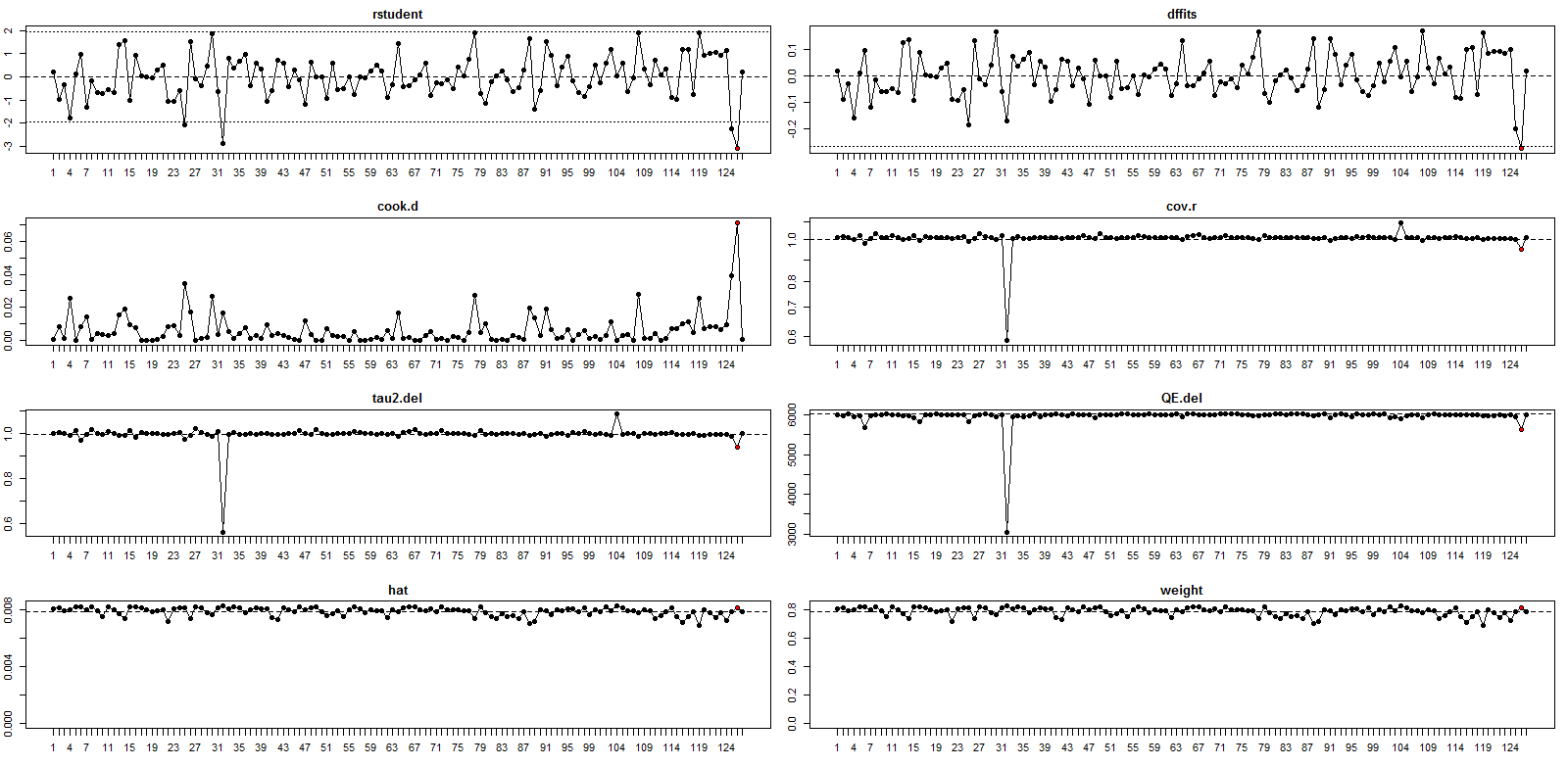
**

**Supplementary Figure 5. Multi-variable meta-regression for MAFLD prevalence in general population.**

**
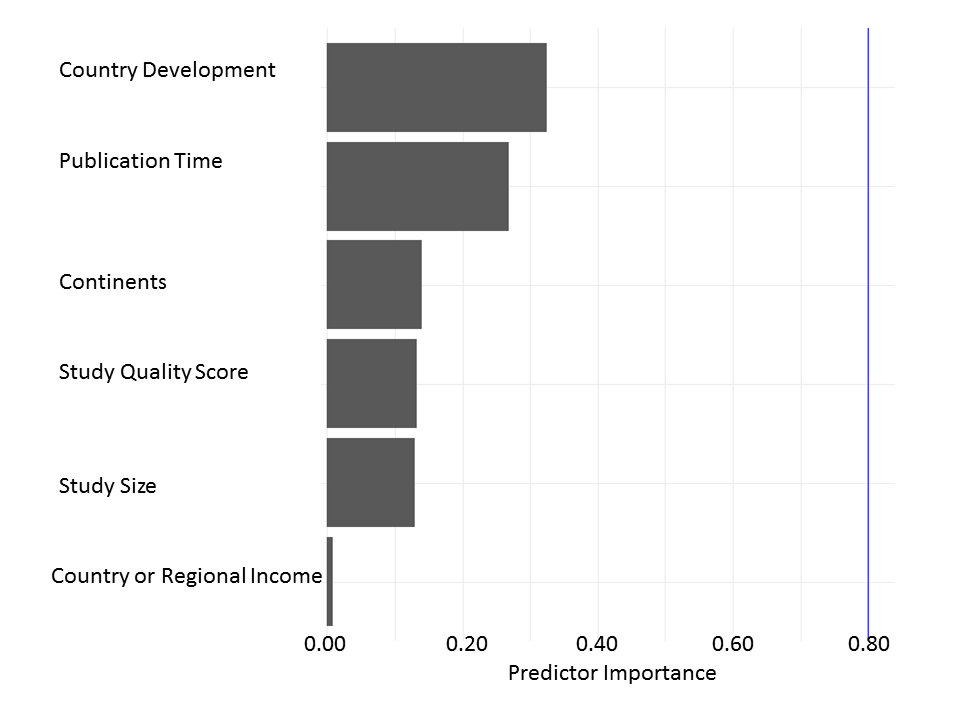
**

**Supplementary Figure 6. Multi-variable meta-regression for MAFLD prevalence in special population.**

**
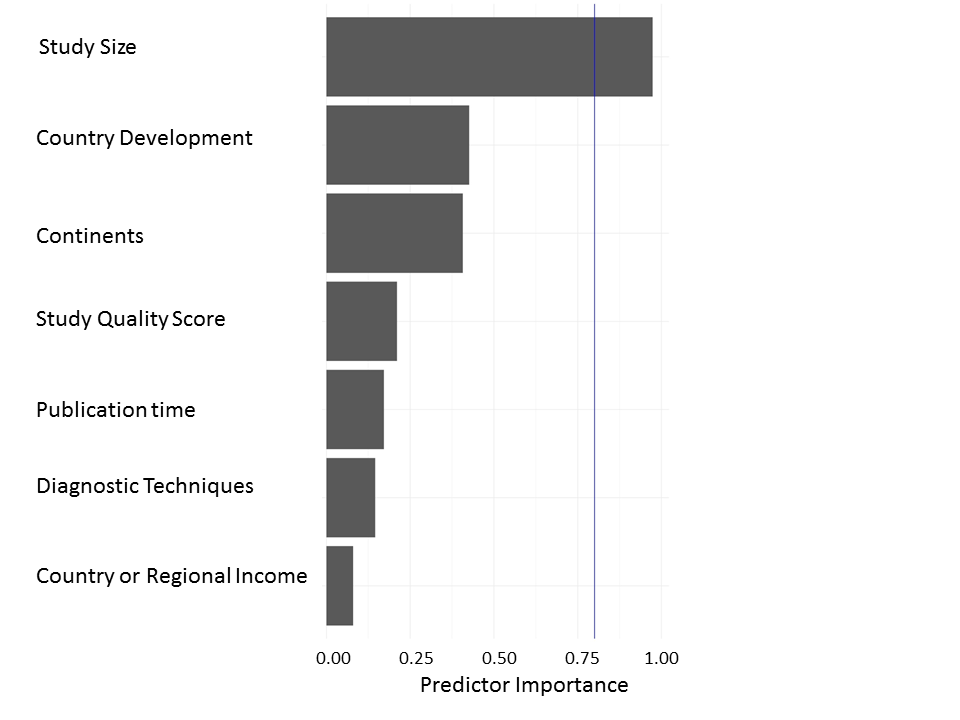
**

**Reference**

1. Adibi A, Kelishadi R, Beihaghi A, Salehi H, Talaei M. Sonographic fatty liver in overweight and obese children, a cross sectional study in Isfahan. *Endokrynol Pol* 2009; **60**(1): 14-9.

2. Alavian SM, Mohammad-Alizadeh AH, Esna-Ashari F, Ardalan G, Hajarizadeh B. Non-alcoholic fatty liver disease prevalence among school-aged children and adolescents in Iran and its association with biochemical and anthropometric measures. *Liver Int* 2009; **29**(2): 159-63.

3. Parray IA. Ultrasonographic Prevalence of Non Alcoholic Fatty Liver Disease (Nafld) in Kashmir Valley School Children *Int J Sci* 2013; **2**(3): 229-301.

4. Pawar SV, Zanwar VG, Choksey AS, et al. Most overweight and obese Indian children have nonalcoholic fatty liver disease. *Ann Hepatol* 2016; **15**(6): 853-61.

5. Geurtsen ML, Santos S, Felix JF, et al. Liver Fat and Cardiometabolic Risk Factors Among School-Age Children. *Hepatology* 2019.

6. Kazemi SA. Assessment of the Relationship Between Prevalence of Reporting Fatty Liver Disease by Ultrasound and Body Mass Index in Children. *Iran J Pediatr* 2017; **27**(1): 1-4.

7. Fu CC, Chen MC, Li YM, Liu TT, Wang LY. The risk factors for ultrasound-diagnosed non-alcoholic fatty liver disease among adolescents. *Ann Acad Med Singapore* 2009; **38**(1): 15-7.

8. Khalkhali HR. Estimating Steatosis Prevalence in Overweight and Obese

Children: Comparison of Bayesian Small Area and Direct

Methods. *Int J Pediatrics* 2016; **4**(9): 3391-97.

9. Lin MS, Lin TH, Guo SE, et al. Waist-to-height ratio is a useful index for nonalcoholic fatty liver disease in children and adolescents: a secondary data analysis. *BMC Public Health* 2017; **17**(1): 851.

10. Lin YC, Chang PF, Chang MH, Ni YH. A common variant in the peroxisome proliferator-activated receptor-gamma coactivator-1alpha gene is associated with nonalcoholic fatty liver disease in obese children. *Am J Clin Nutr* 2013; **97**(2): 326-31.

11. Tominaga K, Kurata JH, Chen YK, et al. Prevalence of fatty liver in Japanese children and relationship to obesity. An epidemiological ultrasonographic survey. *Dig Dis Sci* 1995; **40**(9): 2002-9.

12. Monteiro PA, de Moura Mello Antunes B, Silveira LS, Christofaro DGD, Fernandes RA, Freitas IF. Body composition variables as predictors of NAFLD by ultrasound in obese children and adolescents. *BMC Pediatr* 2014; **14**(1).

13. Namakin K. Prevalence of Non-Alcoholic Fatty Liver Disease (NAFLD) and its Clinical Characteristics in Overweight and Obese Children in the South East of Iran, 2017. *Hepat Mon* 2018; **18**(12): e83525.

14. Nier A, Brandt A, Conzelmann IB, Ozel Y, Bergheim I. Non-Alcoholic Fatty Liver Disease in Overweight Children: Role of Fructose Intake and Dietary Pattern. *Nutrients* 2018; **10**(9).

15. Ramzan M. Sonographic Assessment of Hepatic Steatosis (Fatty Liver) in< School Children of Dera Ismail Khan City (NWFP) Pakistan. *Pak J Nutr* 2009; **8**(6): 797-9.

16. Fernandes MT, Ferraro AA, de Azevedo RA, Fagundes Neto U. Metabolic differences between male and female adolescents with non-alcoholic fatty liver disease, as detected by ultrasound. *Acta Paediatr* 2010; **99**(8): 1218-23.

17. Black LJ, Jacoby P, She Ping-Delfos WC, et al. Low serum 25-hydroxyvitamin D concentrations associate with non-alcoholic fatty liver disease in adolescents independent of adiposity. *J Gastroenterol Hepatol* 2014; **29**(6): 1215-22.

18. Walker RW, Sinatra F, Hartiala J, et al. Genetic and clinical markers of elevated liver fat content in overweight and obese Hispanic children. *Obesity (Silver Spring)* 2013; **21**(12): E790-7.

19. Zhang X, Wan Y, Zhang S, et al. Nonalcoholic fatty liver disease prevalence in urban school-aged children and adolescents from the Yangtze River delta region: a cross-sectional study. *Asia Pac J Clin Nutr* 2015; **24**(2): 281-8.

20. Tsuruta G, Tanaka N, Hongo M, et al. Nonalcoholic fatty liver disease in Japanese junior high school students: its prevalence and relationship to lifestyle habits. *J Gastroenterol* 2010; **45**(6): 666-72.

21. Zhao YZ, Gan YG, Zhou JL, et al. Accuracy of multi-echo Dixon sequence in quantification of hepatic steatosis in Chinese children and adolescents. *World J Gastroenterol* 2019; **25**(12): 1513-23.

22. Dai D, Wen F, Zhou S, et al. Association of MTTP gene variants with pediatric NAFLD: A candidate-gene-based analysis of single nucleotide variations in obese children. *PLoS One* 2017; **12**(9): e0185396.

23. Huang SC, Yang YJ. Serum retinol-binding protein 4 is independently associated with pediatric NAFLD and fasting triglyceride level. *J Pediatr Gastroenterol Nutr* 2013; **56**(2): 145-50.

24. Kim IK, Kim J, Kang JH, Song J. Serum leptin as a predictor of fatty liver in 7-year-old Korean children. *Ann Nutr Metab* 2008; **53**(2): 109-16.

25. Lin YC, Chang PF, Hu FC, Chang MH, Ni YH. Variants in the UGT1A1 gene and the risk of pediatric nonalcoholic fatty liver disease. *Pediatrics* 2009; **124**(6): e1221-7.

26. Monteiro PA, Mota J, Silveira LS, et al. Morphological and metabolic determinants of nonalcoholic fatty liver disease in obese youth: a pilot study. *BMC Res Notes* 2013; **6**: 89.

27. Berna-Serna JD, Sanchez-Jimenez R, Velazquez-Marin F, et al. Acoustic radiation force impulse imaging for detection of liver fibrosis in overweight and obese children. *Acta Radiol* 2018; **59**(2): 247-53.

28. Shashaj B, Bedogni G, Graziani MP, et al. Origin of cardiovascular risk in overweight preschool children: a cohort study of cardiometabolic risk factors at the onset of obesity. *JAMA Pediatr* 2014; **168**(10): 917-24.

29. Kao JT, Wang JH, Hung CH, et al. Changing aetiology of liver dysfunction in the new generation of a hepatitis B and C-endemic area: cross-sectional studies on adolescents born in the first 10 years after universal hepatitis B vaccination. *Liver Int* 2008; **28**(9): 1298-304.

30. Akcam M, Boyaci A, Pirgon O, Koroglu M, Dundar BN. Importance of the liver ultrasound scores in pubertal obese children with nonalcoholic fatty liver disease. *Clin Imaging* 2013; **37**(3): 504-8.

31. Alp H, Karaarslan S, Selver Eklioglu B, Atabek ME, Altin H, Baysal T. Association between nonalcoholic fatty liver disease and cardiovascular risk in obese children and adolescents. *Can J Cardiol* 2013; **29**(9): 1118-25.

32. Arenaza L, Medrano M, Oses M, et al. Dietary determinants of hepatic fat content and insulin resistance in overweight/obese children: a cross-sectional analysis of the Prevention of Diabetes in Kids (PREDIKID) study. *Br J Nutr* 2019; **121**(10): 1158-65.

33. Arslan N, Buyukgebiz B, Ozturk Y, Cakmakci H. Fatty liver in obese children: prevalence and correlation with anthropometric measurements and hyperlipidemia. *Turk J Pediatr* 2005; **47**(1): 23-7.

34. Boyraz M, Hatipoglu N, Sari E, et al. Non-alcoholic fatty liver disease in obese children and the relationship between metabolic syndrome criteria. *Obes Res Clin Pract* 2014; **8**(4): e356-63.

35. Fu JF, Shi HB, Liu LR, et al. Non-alcoholic fatty liver disease: An early mediator predicting metabolic syndrome in obese children? *World J Gastroenterol* 2011; **17**(6): 735-42.

36. Atabek ME, Selver Eklioglu B, Akyurek N. Which metabolic syndrome criteria best predict non-alcoholic fatty liver disease in children? *Eat Weight Disord* 2014; **19**(4): 495-501.

37. Bedogni G, Gastaldelli A, Manco M, et al. Relationship between fatty liver and glucose metabolism: a cross-sectional study in 571 obese children. *Nutr Metab Cardiovasc Dis* 2012; **22**(2): 120-6.

38. Belei O, Olariu L, Dobrescu A, Marcovici T, Marginean O. The relationship between non-alcoholic fatty liver disease and small intestinal bacterial overgrowth among overweight and obese children and adolescents. *J Pediatr Endocrinol Metab* 2017; **30**(11): 1161-8.

39. Benetolo PO, Fernandes MIM, Ciampo I, Elias-Junior J, Sawamura R. Evaluation of nonalcoholic fatty liver disease using magnetic resonance in obese children and adolescents. *J Pediatr (Rio J)* 2019; **95**(1): 34-40.

40. Burgert TS, Taksali SE, Dziura J, et al. Alanine aminotransferase levels and fatty liver in childhood obesity: associations with insulin resistance, adiponectin, and visceral fat. *J Clin Endocrinol Metab* 2006; **91**(11): 4287-94.

41. Cardoso AS, Gonzaga NC, Medeiros CC, Carvalho DF. Association of uric acid levels with components of metabolic syndrome and non-alcoholic fatty liver disease in overweight or obese children and adolescents. *J Pediatr (Rio J)* 2013; **89**(4): 412-8.

42. Chan DF, Li AM, Chu WC, et al. Hepatic steatosis in obese Chinese children. *Int J Obes Relat Metab Disord* 2004; **28**(10): 1257-63.

43. Daar G, Serin HI, Ede H, Husrevsahi H. Association between the corrected QT interval, carotid artery intima-media thickness, and hepatic steatosis in obese children. *Anatol J Cardiol* 2016; **16**(7): 524-8.

44. Das MK, Bhatia V, Sibal A, et al. Prevalence of Nonalcoholic Fatty Liver Disease in Normal-weight and Overweight Preadolescent Children in Haryana, India. *Indian Pediatr* 2017; **54**(12): 1012-6.

45. Zusi C, Mantovani A, Olivieri F, et al. Contribution of a genetic risk score to clinical prediction of hepatic steatosis in obese children and adolescents. *Dig Liver Dis* 2019; **51**(11): 1586-92.

46. Di Costanzo A, Pacifico L, Chiesa C, et al. Genetic and metabolic predictors of hepatic fat content in a cohort of Italian children with obesity. *Pediatr Res* 2019; **85**(5): 671-7.

47. Dursun F GN, Su Dur SM, Kirmizibekmez H. The relation between vitamin D level and hepatosteatosis in obese children. *North Clin Istanb* 2019; **6**(1): 28-32.

48. el-Karaksy HM, el-Koofy NM, Anwar GM, el-Mougy FM, el-Hennawy A, Fahmy ME. Predictors of non-alcoholic fatty liver disease in obese and overweight Egyptian children: single center study. *Saudi J Gastroenterol* 2011; **17**(1): 40-6.

49. Eminoglu TF, Camurdan OM, Oktar SO, Bideci A, Dalgic B. Factors related to non-alcoholic fatty liver disease in obese children. *Turk J Gastroenterol* 2008; **19**(2): 85-91.

50. Erol M, Bostan Gayret O, Tekin Nacaroglu H, et al. Association of Osteoprotegerin with Obesity, Insulin Resistance and Non-Alcoholic Fatty Liver Disease in Children. *Iran Red Crescent Med J* 2016; **18**(11): e41873.

51. Felix DR, Costenaro F, Gottschall CB, Coral GP. Non-alcoholic fatty liver disease (Nafld) in obese children- effect of refined carbohydrates in diet. *BMC Pediatr* 2016; **16**(1): 187.

52. Ferraioli G, Calcaterra V, Lissandrin R, et al. Noninvasive assessment of liver steatosis in children: the clinical value of controlled attenuation parameter. *BMC Gastroenterol* 2017; **17**(1): 61.

53. Fonvig CE, Chabanova E, Andersson EA, et al. 1H-MRS Measured Ectopic Fat in Liver and Muscle in Danish Lean and Obese Children and Adolescents. *PLoS One* 2015; **10**(8): e0135018.

54. Gheibi S. Prevalence and Predictors of Non-Alcoholic Fatty Liver Disease in Obese and Overweight Children in the Northwest of Iran. *Hepat Mon* 2019; **19**(10): e92199.

55. Hamza RT, Elfaramawy AA, Mahmoud NH. Serum Pentraxin 3 Fragment as a Noninvasive Marker of Nonalcoholic Fatty Liver Disease in Obese Children and Adolescents. *Horm Res Paediatr* 2016; **86**(1): 11-20.

56. Han X, Xu P, Zhou J, Liu Y, Xu H. Fasting C-peptide is a significant indicator of nonalcoholic fatty liver disease in obese children. *Diabetes Res Clin Pract* 2020; **160**: 108027.

57. Hatipoglu N, Dogan S, Mazicioglu MM, Kurtoglu S. Relationship between Neck Circumference and Non-Alcoholic Fatty Liver Disease in Childhood Obesity. *J Clin Res Pediatr Endocrinol* 2016; **8**(1): 32-9.

58. Hu W, Wang M, Yin C, Li S, Liu Y, Xiao Y. Serum complement factor 5a levels are associated with nonalcoholic fatty liver disease in obese children. *Acta Paediatr* 2018; **107**(2): 322-7.

59. Jimenez-Rivera C, Hadjiyannakis S, Davila J, et al. Prevalence and risk factors for non-alcoholic fatty liver in children and youth with obesity. *BMC Pediatr* 2017; **17**(1): 113.

60. Kaltenbach TE, Graeter T, Oeztuerk S, et al. Thyroid dysfunction and hepatic steatosis in overweight children and adolescents. *Pediatr Obes* 2017; **12**(1): 67-74.

61. Wiegand S, Keller KM, Robl M, et al. Obese boys at increased risk for nonalcoholic liver disease: evaluation of 16,390 overweight or obese children and adolescents. *Int J Obes (Lond)* 2010; **34**(10): 1468-74.

62. Zhang HX, Xu XQ, Fu JF, Lai C, Chen XF. Predicting hepatic steatosis and liver fat content in obese children based on biochemical parameters and anthropometry. *Pediatr Obes* 2015; **10**(2): 112-7.

63. Kim JY, Cho J, Yang HR. Biochemical Predictors of Early Onset Non-Alcoholic Fatty Liver Disease in Young Children with Obesity. *J Korean Med Sci* 2018; **33**(16): e122.

64. Kistler KD, Molleston J, Unalp A, et al. Symptoms and quality of life in obese children and adolescents with non-alcoholic fatty liver disease. *Aliment Pharmacol Ther* 2010; **31**(3): 396-406.

65. Kodhelaj K, Resuli B, Petrela E, Malaj V, Jaze H. Non-alcoholic fatty liver disease and non-alcoholic steatohepatitis in Albanian overweight children. *Minerva Pediatr* 2014; **66**(1): 23-30.

66. Labayen I, Ruiz JR, Arenaza L, et al. Hepatic fat content and bone mineral density in children with overweight/obesity. *Pediatr Res* 2018; **84**(5): 684-8.

67. Lee JH, Jeong SJ. What is the appropriate strategy for diagnosing NAFLD using ultrasonography in obese children? *World J Pediatr* 2017; **13**(3): 248-54.

68. Liang S, Cheng X, Hu Y, Song R, Li G. Insulin-like growth factor 1 and metabolic parameters are associated with nonalcoholic fatty liver disease in obese children and adolescents. *Acta Paediatr* 2017; **106**(2): 298-303.

69. Mameli C, Krakauer NY, Krakauer JC, et al. The association between a body shape index and cardiovascular risk in overweight and obese children and adolescents. *PLoS One* 2018; **13**(1): e0190426.

70. de Piano A, Prado WL, Caranti DA, et al. Metabolic and nutritional profile of obese adolescents with nonalcoholic fatty liver disease. *J Pediatr Gastroenterol Nutr* 2007; **44**(4): 446-52.

71. Mohamed RZ, Jalaludin MY, Anuar Zaini A. Predictors of non-alcoholic fatty liver disease (NAFLD) among children with obesity. *J Pediatr Endocrinol Metab* 2020; **33**(2): 247-53.

72. Taghavi Ardakani A SM, Kheirkhah D. Fatty liver disease in obese children in Kashan, Iran. *Caspian J of Pediatr* 2015; **1**(1): 17-21.

73. Navarro-Jarabo JM, Ubina-Aznar E, Tapia-Ceballos L, et al. Hepatic steatosis and severity-related factors in obese children. *J Gastroenterol Hepatol* 2013; **28**(9): 1532-8.

74. Franzese A, Vajro P, Argenziano A, et al. Liver involvement in obese children. Ultrasonography and liver enzyme levels at diagnosis and during follow-up in an Italian population. *Dig Dis Sci* 1997; **42**(7): 1428-32.

75. Guzzaloni G, Grugni G, Minocci A, Moro D, Morabito F. Liver steatosis in juvenile obesity: correlations with lipid profile, hepatic biochemical parameters and glycemic and insulinemic responses to an oral glucose tolerance test. *Int J Obes Relat Metab Disord* 2000; **24**(6): 772-6.

76. Oh MS, Kim S, Lee J, Lee MS, Kim YJ, Kang KS. Factors associated with Advanced Bone Age in Overweight and Obese Children. *Pediatr Gastroenterol Hepatol Nutr* 2020; **23**(1): 89-97.

77. Ozhan B, Ersoy B, Ozkol M, Kiremitci S, Ergin A. Waist to height ratio: a simple screening tool for nonalcoholic fatty liver disease in obese children. *Turk J Pediatr* 2016; **58**(5): 518-23.

78. Pacifico L, Bonci E, Andreoli GM, et al. The Impact of Nonalcoholic Fatty Liver Disease on Renal Function in Children with Overweight/Obesity. *Int J Mol Sci* 2016; **17**(8).

79. Papandreou D, Karabouta Z, Rousso I. Are dietary cholesterol intake and serum cholesterol levels related to nonalcoholic Fatty liver disease in obese children? *Cholesterol* 2012; **2012**: 572820.

80. Pozzato C, Radaelli G, Dall'Asta C, et al. MRI in identifying hepatic steatosis in obese children and relation to ultrasonography and metabolic findings. *J Pediatr Gastroenterol Nutr* 2008; **47**(4): 493-9.

81. Sagi R, Reif S, Neuman G, Webb M, Phillip M, Shalitin S. Nonalcoholic fatty liver disease in overweight children and adolescents. *Acta Paediatr* 2007; **96**(8): 1209-13.

82. Prokopowicz Z. Predictive Value of Adiposity Level, Metabolic Syndrome, and Insulin Resistance for the Risk of Nonalcoholic Fatty Liver Disease Diagnosis in Obese Children. *CAN J GASTROENTEROL* 2018; **2018**: 1-8.

83. Radetti G, Kleon W, Stuefer J, Pittschieler K. Non-alcoholic fatty liver disease in obese children evaluated by magnetic resonance imaging. *Acta Paediatr* 2006; **95**(7): 833-7.

84. Ruiz-Extremera A, Carazo A, Salmeron A, et al. Factors associated with hepatic steatosis in obese children and adolescents. *J Pediatr Gastroenterol Nutr* 2011; **53**(2): 196-201.

85. Schlieske C, Denzer C, Wabitsch M, et al. Sonographically measured suprailiac adipose tissue is a useful predictor of non-alcoholic fatty liver disease in obese children and adolescents. *Pediatr Obes* 2015; **10**(4): 260-6.

86. Damaso AR, do Prado WL, de Piano A, et al. Relationship between nonalcoholic fatty liver disease prevalence and visceral fat in obese adolescents. *Dig Liver Dis* 2008; **40**(2): 132-9.

87. Jung JH, Jung MK, Kim KE, et al. Ultrasound measurement of pediatric visceral fat thickness: correlations with metabolic and liver profiles. *Ann Pediatr Endocrinol Metab* 2016; **21**(2): 75-80.

88. Sezer OB, Bulus D, Hizli S, Andiran N, Yilmaz D, Ramadan SU. Low 25-hydroxyvitamin D level is not an independent risk factor for hepatosteatosis in obese children. *J Pediatr Endocrinol Metab* 2016; **29**(7): 783-8.

89. Yildiz I, Erol OB, Toprak S, et al. Role of vitamin D in children with hepatosteatosis. *J Pediatr Gastroenterol Nutr* 2014; **59**(1): 106-11.

90. Pirgon O, Cekmez F, Bilgin H, Eren E, Dundar B. Low 25-hydroxyvitamin D level is associated with insulin sensitivity in obese adolescents with non-alcoholic fatty liver disease. *Obes Res Clin Pract* 2013; **7**(4): e275-83.

91. Cali AM, Zern TL, Taksali SE, et al. Intrahepatic fat accumulation and alterations in lipoprotein composition in obese adolescents: a perfect proatherogenic state. *Diabetes Care* 2007; **30**(12): 3093-8.

92. Shi JQ, Shen WX, Wang XZ, Huang K, Zou CC. Relationship Between Immune Parameters and Non-alcoholic Fatty Liver Disease in Obese Children. *Indian Pediatr* 2017; **54**(10): 825-9.

93. Torun E, Aydin S, Gokce S, Ozgen IT, Donmez T, Cesur Y. Carotid intima-media thickness and flow-mediated dilation in obese children with non-alcoholic fatty liver disease. *Turk J Gastroenterol* 2014; **25 Suppl 1**: 92-8.

94. Üstyol A. Association of Serum Triglyceride-to-High-density Lipoprotein Cholesterol Ratio with Insulin Resistance and Non-alcoholic Fatty Liver Disease in Children and Adolescents. *Med Bull Hasek* 2017; **55**: 286-91.

95. Vakilishahrbabaki H-S. Association between Non-Alcoholic Fatty Liver Disease and Carotid Intima-Media Thickness in Overweight and Obese Children *J res med dent sci* 2018; **6**(3): 313-8.

96. Trico D, Caprio S, Rosaria Umano G, et al. Metabolic Features of Nonalcoholic Fatty Liver (NAFL) in Obese Adolescents: Findings From a Multiethnic Cohort. *Hepatology* 2018; **68**(4): 1376-90.

97. Wang R, Yang F, Qing L, Huang R, Liu Q, Li X. Decreased serum neuregulin 4 levels associated with non-alcoholic fatty liver disease in children with obesity. *Clin Obes* 2019; **9**(1): e12289.

98. Yang HR, Chang EJ. Insulin resistance, body composition, and fat distribution in obese children with nonalcoholic fatty liver disease. *Asia Pac J Clin Nutr* 2016; **25**(1): 126-33.

99. Silveira LS, Monteiro PA, Antunes Bde M, et al. Intra-abdominal fat is related to metabolic syndrome and non-alcoholic fat liver disease in obese youth. *BMC Pediatr* 2013; **13**: 115.

100. Wasilewska N, Bobrus-Chociej A, Harasim-Symbor E, et al. Increased serum concentration of ceramides in obese children with nonalcoholic fatty liver disease. *Lipids Health Dis* 2018; **17**(1): 216.

101. Yang HR, Yi DY, Choi HS. Comparison between a pediatric health promotion center and a pediatric obesity clinic in detecting metabolic syndrome and non-alcoholic fatty liver disease in children. *J Korean Med Sci* 2014; **29**(12): 1672-7.

102. Gronbaek H, Lange A, Birkebaek NH, et al. Effect of a 10-week weight loss camp on fatty liver disease and insulin sensitivity in obese Danish children. *J Pediatr Gastroenterol Nutr* 2012; **54**(2): 223-8.

103. Saad V, Wicklow B, Wittmeier K, et al. A clinically relevant method to screen for hepatic steatosis in overweight adolescents: a cross sectional study. *BMC Pediatr* 2015; **15**: 151.

104. Zou CC, Liang L, Hong F, Fu JF, Zhao ZY. Serum adiponectin, resistin levels and non-alcoholic fatty liver disease in obese children. *Endocr J* 2005; **52**(5): 519-24.

105. Bohte AE, Koot BG, van der Baan-Slootweg OH, et al. US cannot be used to predict the presence or severity of hepatic steatosis in severely obese adolescents. *Radiology* 2012; **262**(1): 327-34.

106. Chiloiro M, Riezzo G, Chiarappa S, et al. Relationship among fatty liver, adipose tissue distribution and metabolic profile in moderately obese children: an ultrasonographic study. *Curr Pharm Des* 2008; **14**(26): 2693-8.

107. de Silva KS, Wickramasinghe VP, Gooneratne IN. Metabolic consequences of childhood obesity--a preliminary report. *Ceylon Med J* 2006; **51**(3): 105-9.

108. Denzer C, Thiere D, Muche R, et al. Gender-specific prevalences of fatty liver in obese children and adolescents: roles of body fat distribution, sex steroids, and insulin resistance. *J Clin Endocrinol Metab* 2009; **94**(10): 3872-81.

109. Hacihamdioglu B, Okutan V, Yozgat Y, et al. Abdominal obesity is an independent risk factor for increased carotid intima- media thickness in obese children. *Turk J Pediatr* 2011; **53**(1): 48-54.

110. Kim JS, Le KA, Mahurkar S, Davis JN, Goran MI. Influence of elevated liver fat on circulating adipocytokines and insulin resistance in obese Hispanic adolescents. *Pediatr Obes* 2012; **7**(2): 158-64.

111. El-Koofy NM, Anwar GM, El-Raziky MS, et al. The association of metabolic syndrome, insulin resistance and non-alcoholic fatty liver disease in overweight/obese children. *Saudi J Gastroenterol* 2012; **18**(1): 44-9.

112. Ozkol M, Ersoy B, Kasirga E, Taneli F, Bostanci IE, Ozhan B. Metabolic predictors for early identification of fatty liver using doppler and B-mode ultrasonography in overweight and obese adolescents. *Eur J Pediatr* 2010; **169**(11): 1345-52.

113. Papandreou D, Rousso I, Malindretos P, et al. Are saturated fatty acids and insulin resistance associated with fatty liver in obese children? *Clin Nutr* 2008; **27**(2): 233-40.

114. Perseghin G, Bonfanti R, Magni S, et al. Insulin resistance and whole body energy homeostasis in obese adolescents with fatty liver disease. *Am J Physiol Endocrinol Metab* 2006; **291**(4): E697-703.

115. Reinehr T, Roth CL. Fetuin-A and its relation to metabolic syndrome and fatty liver disease in obese children before and after weight loss. *J Clin Endocrinol Metab* 2008; **93**(11): 4479-85.

116. Tock L, Prado WL, Caranti DA, et al. Nonalcoholic fatty liver disease decrease in obese adolescents after multidisciplinary therapy. *Eur J Gastroenterol Hepatol* 2006; **18**(12): 1241-5.

117. Xanthakos S, Miles L, Bucuvalas J, Daniels S, Garcia V, Inge T. Histologic spectrum of nonalcoholic fatty liver disease in morbidly obese adolescents. *Clin Gastroenterol Hepatol* 2006; **4**(2): 226-32.

118. Boza C, Viscido G, Salinas J, Crovari F, Funke R, Perez G. Laparoscopic sleeve gastrectomy in obese adolescents: results in 51 patients. *Surg Obes Relat Dis* 2012; **8**(2): 133-7; discussion 7-9.

119. Koot BG, van der Baan-Slootweg OH, Tamminga-Smeulders CL, et al. Lifestyle intervention for non-alcoholic fatty liver disease: prospective cohort study of its efficacy and factors related to improvement. *Arch Dis Child* 2011; **96**(7): 669-74.

120. Alqahtani A, Elahmedi M, Alswat K, Arafah M, Fagih M, Lee J. Features of nonalcoholic steatohepatitis in severely obese children and adolescents undergoing sleeve gastrectomy. *Surg Obes Relat Dis* 2017; **13**(9): 1599-609.

121. Bobrus-Chociej A, Flisiak-Jackiewicz M, Daniluk U, et al. Estimation of gamma-glutamyl transferase as a suitable simple biomarker of the cardiovascular risk in children with non-alcoholic fatty liver disease. *Acta Biochim Pol* 2018; **65**(4): 539-44.

122. Deeb A, Attia S, Mahmoud S, Elhaj G, Elfatih A. Dyslipidemia and Fatty Liver Disease in Overweight and Obese Children. *J Obes* 2018; **2018**: 8626818.

123. Assuncao SNF, Sorte N, Alves CAD, Mendes PSA, Alves CRB, Silva LR. Inflammatory cytokines and non-alcoholic fatty liver disease (NAFLD) in obese children and adolescents. *Nutr Hosp* 2018; **35**(1): 78-83.

124. Pooja G. Nutritional assessment in obese children with and without non-alcoholic fatty liver disease (NAFLD) in an Urban Area of Punjab, India. *Indian J Public Health De* 2018; **9**(12): 201-7.

125. Kirel B. Nonalcoholic fatty liver diseases in obese children and adolescents. *Turk Arch Ped* 2012; **47**: 172-8.

126. Di Martino M, Pacifico L, Bezzi M, et al. Comparison of magnetic resonance spectroscopy, proton density fat fraction and histological analysis in the quantification of liver steatosis in children and adolescents. *World J Gastroenterol* 2016; **22**(39): 8812-9.

127. Yu EL, Golshan S, Harlow KE, et al. Prevalence of Nonalcoholic Fatty Liver Disease in Children with Obesity. *J Pediatr* 2019; **207**: 64-70.

128. Szybowska P, Wojcik M, Starzyk JB, Sztefko K. Enhanced liver fibrosis (ELF) test in obese children with ultrasound-proven liver steatosis. *Neuro Endocrinol Lett* 2015; **36**(7): 700-5.

129. Pena-Velez R, Garibay-Nieto N, Cal YM-VM, et al. Association between neck circumference and non-alcoholic fatty liver disease in Mexican children and adolescents with obesity. *J Pediatr Endocrinol Metab* 2020; **33**(2): 205-13.

130. Elkabany ZA, Hamza RT, Ismail EAR, et al. Serum visfatin level as a noninvasive marker for nonalcoholic fatty liver disease in children and adolescents with obesity: relation to transient elastography with controlled attenuation parameter. *Eur J Gastroenterol Hepatol* 2019.

131. Ozsu E, Yazicioglu B. Obese boys with low concentrations of high-density lipoprotein cholesterol are at greater risk of hepatosteatosis. *Hormones (Athens)* 2019; **18**(4): 477-84.

132. Hua MC, Huang JL, Hu CC, Yao TC, Lai MW. Including Fibroblast Growth Factor-21 in Combined Biomarker Panels Improves Predictions of Liver Steatosis Severity in Children. *Front Pediatr* 2019; **7**: 420.

133. Di Bonito P, Valerio G, Licenziati MR, et al. High uric acid, reduced glomerular filtration rate and non-alcoholic fatty liver in young people with obesity. *J Endocrinol Invest* 2020; **43**(4): 461-8.

134. Jain V, Kumar A, Ahmad N, et al. Genetic polymorphisms associated with obesity and non-alcoholic fatty liver disease in Asian Indian adolescents. *J Pediatr Endocrinol Metab* 2019; **32**(7): 749-58.

135. Villanueva-Ortega E, Garces-Hernandez MJ, Herrera-Rosas A, et al. Gender-specific differences in clinical and metabolic variables associated with NAFLD in a Mexican pediatric population. *Ann Hepatol* 2019; **18**(5): 693-700.

136. Draijer LG, Feddouli S, Bohte AE, et al. Comparison of diagnostic accuracy of screening tests ALT and ultrasound for pediatric non-alcoholic fatty liver disease. *Eur J Pediatr* 2019; **178**(6): 863-70.

137. Kurku H, Atar M, Pirgon O, et al. Pubertal Status and Gonadal Functions in Obese Boys with Fatty Liver. *Metab Syndr Relat Disord* 2019; **17**(2): 102-7.

138. Ramirez-Velez R, Izquierdo M, Correa-Bautista JE, et al. Liver Fat Content and Body Fat Distribution in Youths with Excess Adiposity. *J Clin Med* 2018; **7**(12).

139. Flisiak-Jackiewicz M, Bobrus-Chociej A, Tarasow E, Wojtkowska M, Bialokoz-Kalinowska I, Lebensztejn DM. Predictive Role of Interleukin-18 in Liver Steatosis in Obese Children. *Can J Gastroenterol Hepatol* 2018; **2018**: 3870454.

140. Bacha F, Tomsa A, Bartz SK, et al. Nonalcoholic Fatty Liver Disease in Hispanic Youth With Dysglycemia: Risk for Subclinical Atherosclerosis? *J Endocr Soc* 2017; **1**(8): 1029-40.

141. Balanescu A, Balanescu P, Comanici V, et al. Lipid profile pattern in pediatric overweight population with or without NAFLD in relation to IDF criteria for metabolic syndrome: a preliminary study. *Rom J Intern Med* 2018; **56**(1): 47-54.

142. Lu LP, Wan YP, Xun PC, et al. Serum bile acid level and fatty acid composition in Chinese children with non-alcoholic fatty liver disease. *J Dig Dis* 2017; **18**(8): 461-71.

143. Chabanova E, Fonvig CE, Bojsoe C, Holm JC, Thomsen HS. (1)H MRS Assessment of Hepatic Fat Content: Comparison Between Normal- and Excess-weight Children and Adolescents. *Acad Radiol* 2017; **24**(8): 982-7.

144. Lee S, Kuk JL. Visceral fat is associated with the racial differences in liver fat between black and white adolescent boys with obesity. *Pediatr Diabetes* 2017; **18**(7): 660-3.

145. Sheldon RD, Kanosky KM, Wells KD, et al. Transcriptomic differences in intra-abdominal adipose tissue in extremely obese adolescents with different stages of NAFLD. *Physiol Genomics* 2016; **48**(12): 897-911.

146. Jin R, Le NA, Cleeton R, et al. Amount of hepatic fat predicts cardiovascular risk independent of insulin resistance among Hispanic-American adolescents. *Lipids Health Dis* 2015; **14**: 39.

147. Chang PF, Lin YC, Liu K, Yeh SJ, Ni YH. Heme oxygenase-1 gene promoter polymorphism and the risk of pediatric nonalcoholic fatty liver disease. *Int J Obes (Lond)* 2015; **39**(8): 1236-40.

148. Klusek-Oksiuta M, Bialokoz-Kalinowska I, Tarasow E, Wojtkowska M, Werpachowska I, Lebensztejn DM. Chemerin as a novel non-invasive serum marker of intrahepatic lipid content in obese children. *Ital J Pediatr* 2014; **40**: 84.

149. O'Sullivan TA, Oddy WH, Bremner AP, et al. Lower fructose intake may help protect against development of nonalcoholic fatty liver in adolescents with obesity. *J Pediatr Gastroenterol Nutr* 2014; **58**(5): 624-31.

150. Santoro N, Caprio S, Giannini C, et al. Oxidized fatty acids: A potential pathogenic link between fatty liver and type 2 diabetes in obese adolescents? *Antioxid Redox Signal* 2014; **20**(2): 383-9.

151. Lebensztejn DM, Kowalczuk D, Tarasow E, Skiba E, Kaczmarski M. Tumor necrosis factor alpha and its soluble receptors in obese children with NAFLD. *Adv Med Sci* 2010; **55**(1): 74-9.

152. Demircioglu F, Kocyigit A, Arslan N, Cakmakci H, Hizli S, Sedat AT. Intima-media thickness of carotid artery and susceptibility to atherosclerosis in obese children with nonalcoholic fatty liver disease. *J Pediatr Gastroenterol Nutr* 2008; **47**(1): 68-75.

153. Love-Osborne KA, Nadeau KJ, Sheeder J, Fenton LZ, Zeitler P. Presence of the metabolic syndrome in obese adolescents predicts impaired glucose tolerance and nonalcoholic fatty liver disease. *J Adolesc Health* 2008; **42**(6): 543-8.

154. Shalitin S, Phillip M. Frequency of cardiovascular risk factors in obese children and adolescents referred to a tertiary care center in Israel. *Horm Res* 2008; **69**(3): 152-9.

155. Nichols PH, Pan Y, May B, et al. Effect of TSH on Non-Alcoholic Fatty Liver Disease (NAFLD) independent of obesity in children of predominantly Hispanic/Latino ancestry by causal mediation analysis. *PLoS One* 2020; **15**(6): e0234985.

156. Barretto JR, Boa-Sorte N, Vinhaes CL, et al. Heightened Plasma Levels of Transforming Growth Factor Beta (TGF-beta) and Increased Degree of Systemic Biochemical Perturbation Characterizes Hepatic Steatosis in Overweight Pediatric Patients: A Cross-Sectional Study. *Nutrients* 2020; **12**(6).
